# How people know their risk preference

**DOI:** 10.1038/s41598-020-72077-5

**Published:** 2020-09-21

**Authors:** Ruben C. Arslan, Martin Brümmer, Thomas Dohmen, Johanna Drewelies, Ralph Hertwig, Gert G. Wagner

**Affiliations:** 1 Center for Adaptive Rationality, Max Planck Institute for Human Development, Lentzeallee 94, 14195 Berlin, Germany; 2 University of Leipzig, Leipzig, Germany; 3 Institute for Applied Microeconomics, University of Bonn, Bonn, Germany; 4 Institute of Labor Economics (IZA), Bonn, Germany; 5 Maastricht University, Maastricht, The Netherlands; 6 German Institute for Economic Research, Berlin, Germany; 7 CESifo GmbH, Munich, Germany; 8 Humboldt University of Berlin, Berlin, Germany

**Keywords:** Psychology, Human behaviour

## Abstract

People differ in their willingness to take risks. Recent work found that revealed preference tasks (e.g., laboratory lotteries)—a dominant class of measures—are outperformed by survey-based stated preferences, which are more stable and predict real-world risk taking across different domains. How can stated preferences, often criticised as inconsequential “cheap talk,” be more valid and predictive than controlled, incentivized lotteries? In our multimethod study, over 3,000 respondents from population samples answered a single widely used and predictive risk-preference question. Respondents then explained the reasoning behind their answer. They tended to recount diagnostic behaviours and experiences, focusing on voluntary, consequential acts and experiences from which they seemed to infer their risk preference. We found that third-party readers of respondents’ brief memories and explanations reached similar inferences about respondents’ preferences, indicating the intersubjective validity of this information. Our results help unpack the self perception behind stated risk preferences that permits people to draw upon their own understanding of what constitutes diagnostic behaviours and experiences, as revealed in high-stakes situations in the real world.

## Introduction

Consequential decisions about health, finances, and relationships often invoke the question of how much risk an individual is willing to take. Risk preferences are thus widely studied in experimental economics; personality, cognitive, and clinical psychology; and even animal personality research^1–4^. Measures of risk preference can help predict a wide range of behaviours, from smoking and pathological gambling^5^ to self-employment and holding stocks^6–9^.

Two very different measurement traditions have investigated risk preferences in humans. The *revealed preference* approach, common in economics, has sought to study choices under risk in the field^10^ and in the laboratory^11^. The paradigmatic research designs in this tradition are observational studies of real behaviours (e.g., consumption and saving) and controlled choices between monetary lotteries. At the same time, personality and clinical psychologists, as well as some economists, have used a *stated preference* approach in which people are asked to state their willingness to take risks, using either general questions or hypothetical scenarios. Our present goal is to explain why and how stated preferences are informative by embedding them in the literature on self-perception and self-insight. In doing so, we provide insight into how people rely on their experiences to infer their preferences and how this affects our measurements.

Economists have been skeptical about the validity of stated preferences, particularly in situations in which individuals perceive benefits from (un)truthful and self-serving answers (e.g.^12^). Inferring preferences from real-life behaviour is fraught with assumptions, such as temporal stability and adequate control of confounding factors. To verify these assumptions, economists have typically turned to revealed preference measures, which offer greater control over confounding factors while still measuring “real” behaviour (see^13–15^). Ironically, when researchers compared revealed and stated risk preference measures systematically^5,16–18^, they found that the behavioural measures used in the revealed preference approach generally underperformed relative to the stated preference measures in terms of reliability, retest stability, and criterion validity (see Supplement S1 for a more detailed review)^4,13^. The behavioural measures used in the revealed preference approach did not correlate strongly across measures, meaning that they did not capture a clear latent preference that drives behaviour across different choice situations—even when differences between tasks were abstracted away by modelling the decision process^19^. In contrast, the stated risk preferences correlated across measures and suggested the existence of a general risk factor. Finally, convergence between revealed and stated preferences has been found to be low, particularly when third variables like age and gender are kept constant^5,9,20,21^.

While much research has investigated the cognitive processes that underlie behaviour (e.g., choices) in the lab-based revealed preferences approach^19,22^, little is known about the processes that shape responses in the stated preference approach (but see^23,24^). This gap may be another reason why many economists remain skeptical about the stated preference approach. Although self-reports are widely used in psychology, their accuracy is often disputed, with some researchers emphasizing their context sensitivity and potential for bias and self-enhancement^25–27^ and others arguing that self-reports are often valid under real-world conditions^28–32^.

While few researchers would assert that people can draw on absolute, internal values to objectively report their preferences or personality, there is reason to believe that people have a keen sense of where they stand in relation to others on certain dimensions. It has been argued^33^ that people’s self-perception co-opts the abilities used for social perception: The same instant recognition that allows a person to call someone sprinting across a busy street a “crazy bastard”^34^ can also be applied by a person to themself. Social psychologists have focused on explaining how this co-opted adaptation causes lapses in self-judgment^35^, while recent work in personality psychology draws on the concept of self-other knowledge asymmetries to explain why people know themselves better than others do in some but not all areas^30,31^. Such asymmetries may also explain some of the discrepancy in validity between stated and revealed preference measures: People's risk preferences can be “revealed” in their choices and actions, but the very same action—depending on a person’s psychological state, current needs, and overall abilities^36,37^—could be a risk taken willingly, an impulse regretted immediately, a last resort when cornered, or child’s play for the highly skilled. Unlike the decision maker, external observers cannot easily access these internal states to infer the preferences from the observed behaviour.

To unpack the process of self-perception, we investigated how people translate their memories and intuitions into an answer to the question “How do you see yourself: Are you generally a person who is fully prepared to take risks or do you try to avoid taking risks?” on a scale from 0 to 10 (“unwilling to take risks” to “fully prepared to take risks”). This single question, the General Risk Question (GRQ)^6^ has been used in several large and widely analyzed surveys^38–40^. The GRQ is predictive of real-world risk taking^6^ and is one of the best indicators of the general factor of risk preferences^5^. Many genetic loci linked to risk preferences in a genome-wide association study were identified through the use of similar single-item questions^41^.

Here, we took a descriptive approach because systematically varying questions, examples, and reference frames^42–44^ would require deviations from the widely used GRQ. Instead, we let participants speak: we asked people to explain how they answered the GRQ and which risks they thought about in order to illuminate how people infer their own risk preferences from their decisions, indecisions, and regrets. We were interested in three aspects of how people evaluate their risk preferences.

1. What kind of risks do people consider when they judge themselves? Are these concrete everyday risks with clear consequences, or small, cumulative risks with stochastic consequences? Which social and temporal reference frames do people use? And do they mainly think about risks they took and considered worthwhile, or do risks they avoided or regretted taking feature too?
2. Do age and gender affect the risks people invoke and experience?
3. Can independent third parties agree on what people's experiences say about their preferences?

We collected stated risk preferences as part of two large, age-heterogeneous survey studies in Germany: the 2017 interim survey of the BASE-II study^45^ and the 2017/2018 German Socioeconomic Panel Innovation Sample (SOEP-IS)^46^. Across both studies, 3,493 respondents answered the GRQ. After doing so, they were asked to explain their response in closed-form questions about the social and temporal reference frames they had had in mind, as well as in free-text questions about the topics and events they had thought about. In a second free-text question, they listed the biggest risks they had taken in the past year. BASE-II respondents were also asked if the risks they had taken had been worthwhile.

**Figure 1:**
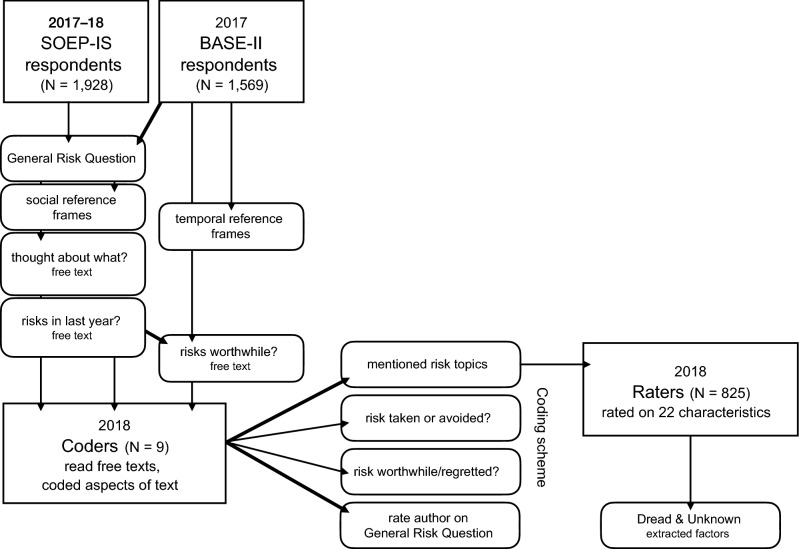
Flow chart of the data collection, coding, and rating steps. Boxes show samples; rounded rectangles reflect steps in the data collecting and processing.

To quantify the topics featured in respondents’ free-text answers, we conducted two further studies (Fig. 1). For one study, we designed a coding scheme with a list of broad risk domains and individual hazards, based on both the extant literature and the free-text responses in this study. A set of coders then read the free-text responses. We used their codings to measure the extent to which there was intersubjective agreement about how risk preferences are revealed in experiences and choices. Specifically, we examined whether coders agreed with each other and with the authors of the text as to whether the risks the authors said they had taken, not taken, or regretted taking validly signal high or low risk preference. Nine coders read approximately 1,000 free-text answers each, so that each answer was coded in triplicate. Coders noted the presence of risk domains, such as investments or health, as well as more specific hazards, such as skydiving or divorce. Finally, each coder estimated—based solely on the available text—the respondent’s stated risk preference (GRQ).

In another study, we aimed to compare the coded risk domains and hazards quantitatively across several characteristics. To this end, participants in an online panel (n = 825) each rated three to five randomly drawn hazards from our coding scheme, ranging from divorce to cycling. They rated each hazard on 20 characteristics (e.g., voluntariness, immediacy) known in the literature^47,48^ and on two additional characteristics that we added to differentiate social from mortality risks. Following Slovic^47^, we extracted the factors Dread and Unknown from 16 of these characteristics in a confirmatory factor analysis (see Supplement S8.2). Dreaded risks tend to be global, uncontrollable, involuntary, and hard to reduce, and people prefer strict regulation against them. Unknown risks tend to be more elusive: they are difficult to observe and their effects are delayed. Both factors feature prominently in the psychometric approach to studying risk perception^47^.

## Results

### What risks do people invoke?

Across both studies, 2,510 respondents (72%) gave free-text responses that were sufficiently elaborate to code risk domains and hazards (see Supplement S5 for an analysis of nonresponse and Supplement S7.3 for an analysis of the elaborateness of responses). The coded topic frequencies for the two free-text questions were highly correlated (*r* = 0.94), so we report summed frequencies in the following (see Supplement S7.1 for separate counts). Table 1 shows the frequency with which risk domains and hazards were mentioned and Supplement S7.2 shows how often certain combinations of domains were mentioned (e.g., career, investment, and relationship risks were often mentioned together).

**Table 1:** Frequencies with which risk domains and hazards were mentioned.

Domain	Mentions	Q1	Hazards
Investments	771	418	Investment (242), bought home (86), founded company (15), sold home (13)
Relationships	760	399	Moving (132), conflicts (79), children: general (59), speaking out (44), separation (36), pregnant (26), marriage (24), moving in (14), divorce (13), colleagues (10), affairs (7), sticking by (7)
Traffic	645	332	Car (278), bicycle (172), motorcycle (44), airplane (33), bus (18), train (1)
Career	612	321	
Safety	437	239	Disregarding own frailty (85), working around house and garden (75), going out alone (36), risking being mugged (34), showing moral courage (31), exposure to terrorism (3), fireworks (0), weapons (0)
Travel	433	212	
Sports	414	233	Mountaineering (100), water sports (36), skiing (33), skydiving (23), swimming (19), bungee jumping (8), jogging (7), motor sports (1), shooting sports (0)
Health	371	136	Surgery (116), drinking (15), immediate health risks: other (14), long-term health risks: other (9), drugs: other (8), sex (7), smoking (7), unhealthy food (7), medication side effects (2), vaccines (1), cannabis (0), GMO food (0), toxins: other (0), pesticides (0), air pollution (0), coffee (0), vaccine avoidance (0)
Other	229	144	
Gambling	119	59	
Crime	37	15	Commit misdemeanour (18), commit crime (4)
Cataclysm	14	10	Terror attack (3), earthquake (1), flooding (0), nuclear waste/war/accidents/fallout (0)

All numbers reflect the number of times a risk domain or hazard was coded from the texts written by our respondents in response to both of the free-text questions. The column Q1 shows the number of mentions in response to the first free-text question (on which risks people thought about).

**Figure 2:**
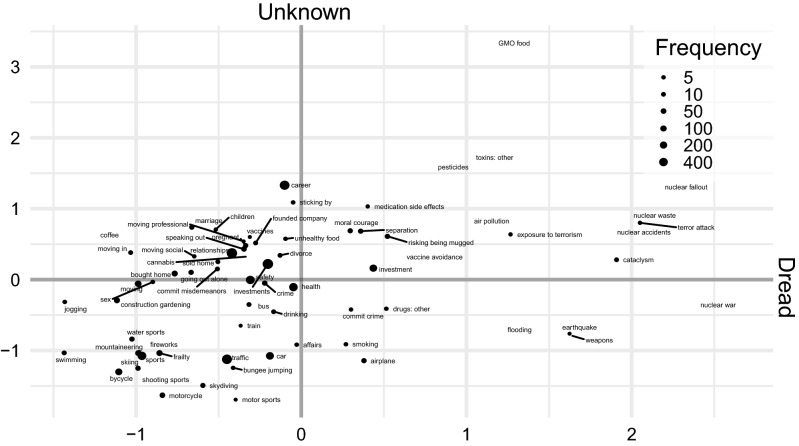
Risk domains and hazards in a coordinate system of the Dread (left to right) and Unknown (bottom to top) factors. Factors were extracted from the risk perception ratings of our online sample and standardised to mean = 0 and SD = 1. The size of the dots reflects how often these risk domains and hazards were coded from the responses to the two free-text questions.

The hazards respondents mentioned frequently tended to be lower on the factors Unknown (Spearman rank-correlation with frequency: *r* = − 0.28) and Dread (*r* = − 0.46). As can be seen in Fig. 2, mentioned risks were more broadly distributed across the Unknown than the Dread factor. In addition to the coded categories, we present unigram and bigram word clouds for all responses in Supplement S7.7.

When thinking about their risk preferences, respondents focused on more common, known hazards. We can further characterize the frequently mentioned hazards in terms of the individual rated characteristics (italicised in the following, see also Supplement S8.3): for example, people tended to frequently reference risks that they took *voluntarily* (*r* = 0.34*,* e.g., sports, as opposed to terror attacks), that had consequences known to those *exposed* (*r* = 0.29, e.g., getting on a ladder, as opposed to side effects from medication), that were old and familiar (*newness, r* = − 0.22) and which they could *control* and *prevent* (*r*s = 0.41, 0.43, e.g., cars and bikes, as opposed to planes and buses).

In line with that pattern, respondents focused on episodic health risks such as surgery and other interventions with immediate consequences (*r* = 0.19), and referred less to risks that have cumulative and delayed effects (e.g., drinking, smoking). The exceptions to these trends were often nonmortality risks such as investment, career, and relationship risks, which do not always have immediate, knowable consequences. In fact, career and education decisions were the highest-ranked risk on the Unknown factor. Nobody mentioned what our online raters identified as the three most unknown hazards: GMO food, pesticides, and “toxins: other”. Respondents almost never mentioned hazards that were dreadful*,* such as nuclear war or similar cataclysmic events. The most common dreadful hazard—terror attacks—was mentioned by only nine respondents.

### Which social and temporal reference frames do people use?

**Figure 3:**
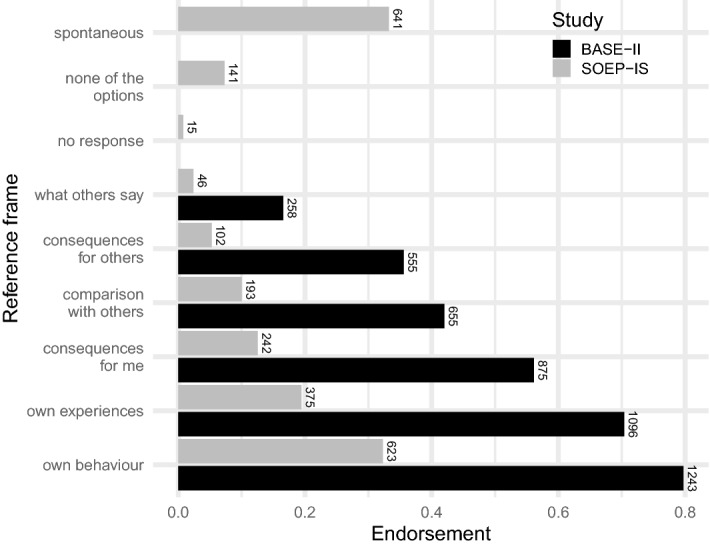
Social reference frames. BASE-II respondents endorsed more options than did SOEP-IS respondents and did not have the option to say they responded spontaneously or based on something else. The options that were common to both studies were similar in rank.

**Figure 4:**
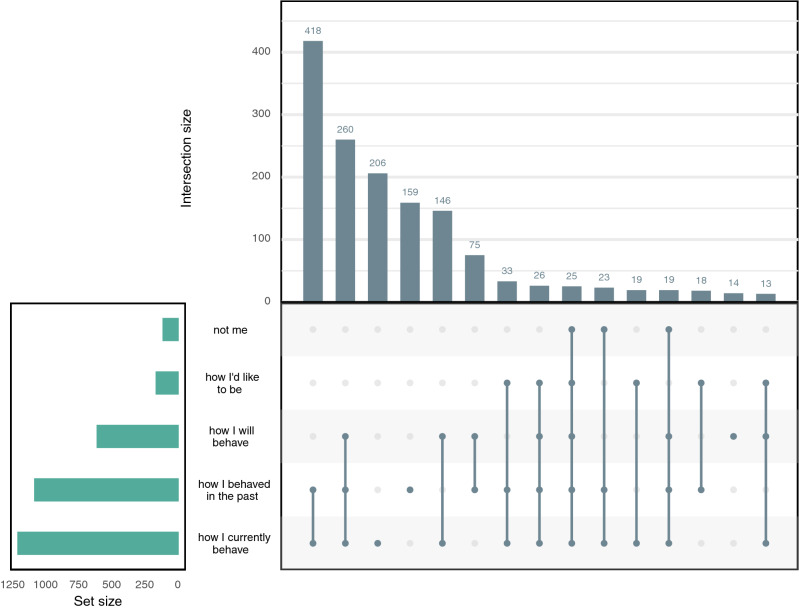
Temporal reference frames. This UpSet plot^49^ shows the frequency of endorsing one or several options in the question about temporal reference frames in the BASE-II study. The lower left panel shows simple counts; the top panel shows how options were combined. Only the 15 most common combinations are shown here.

Respondents reported diverse social and temporal reference frames in our two closed-form questions. In both studies, most respondents stated that they thought of their own experiences and behaviour, or the consequences of their actions, whereas a substantial minority also mentioned comparison with others or what others say (Fig. 3). We varied the available response options across the two samples (see Supplement S6). The BASE-II respondents answered an additional question about temporal reference frames; almost all said they thought about the present (78%, n = 1,209) or the past (70%, n = 1,081), and most of these respondents (52%, n = 807) thought about past and present (Fig. 4). A substantial fraction of respondents (39%, n = 607) also referred to the future, but rarely without thinking about either the past or the present as well (1%, n = 20). Some (10%, n = 161) respondents additionally endorsed an aspirational reference frame—they thought about how they would like to be—or said they did not think about themselves, but these respondents usually endorsed the more common temporal reference frames as well.

### Do people think about risks they took or avoided?

Among those who mentioned codeable risks, most respondents (53%, n = 1,129) clearly mentioned risks they took, and only 2% mentioned risks they avoided. For the remainder of responses, it was unclear whether risks were taken or avoided (32%), no two coders agreed (12%), or respondents wrote about risks that others took (1%). Crime, gambling, and investment risks were mentioned as risks avoided more frequently than the average risk (9%, 3%, and 3%, respectively).

BASE-II respondents were asked whether the risks they had taken in the last year had been worthwhile. Of those respondents who listed a risk taken in the last year, most reported that the risks had been worthwhile (68%, n = 709) or partially worthwhile (11%). A total of 3% gave different answers for different risks, and 4% said it was too soon to tell whether it had been worth taking the risk. Only 9% clearly stated that taking the risk had not been worthwhile, and 1% said they did not know. For 4% of responses no two coders agreed. Compared to the average level of regret, respondents appeared to particularly regret risks taken in the domains of gambling (26% of cases when gambling was the topic), crime (17%), and traffic (14%), whereas few regretted taking risks related to relationships (5%), sports (4%), their career or education (3%), and travel (1%).

### Do age and gender affect the risks people invoke and experience?

**Figure 5:**
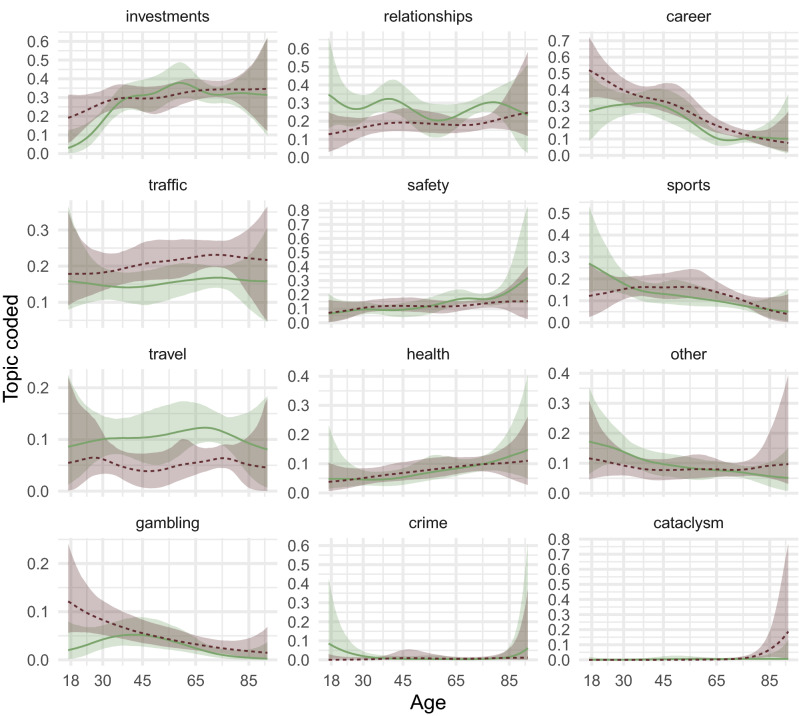
Age trends and gender differences in risk domains coded based on what people thought about when answering the General Risk Question. The lines show regression splines by gender with shaded 95% credible intervals. Solid green lines indicate women; dashed red lines indicate men. The BASE-II and SOEP-IS samples were pooled and a contrast-coded dummy for study was adjusted for. In Supplement S7.4, we report model comparisons to estimate support for age and gender differences, as well as age-by-gender interactions using approximative leave-one-out crossvalidation. Average trends were similar after imputation (see Supplement S7.5).

On average, men were more likely to mention risks of injury such as traffic (95% CI of the difference in proportions in response to Q1: [0.02; 0.09]) and sports risks [− 0.01; 0.05]. Women mentioned relationship [− 0.14; − 0.06] and travel risks [− 0.10; − 0.04] more often, and career risks less often [0.01; 0.08], than men did. Older people—women and men alike—rarely mentioned career and education or sports, but increasingly mentioned traffic, health, and safety risks (Fig. 5; see also Supplement S7.4). Young men were most likely to mention gambling; otherwise age trends were largely parallel for men and women. Age and gender differences were similar for questions 1 and 2 (see Supplements S7.4, Supplement S7.6). Age and gender differences in reference frames were not as pronounced as topic differences, although males reported more often that they referred to their own experiences [0.02; 0.08] and behaviour [0.01; 0.07] and older people were more likely to report that they referred to future, not past events (see Supplement S6).

### Can independent third parties agree on what people's experiences say about their preferences?

**Figure 6:**
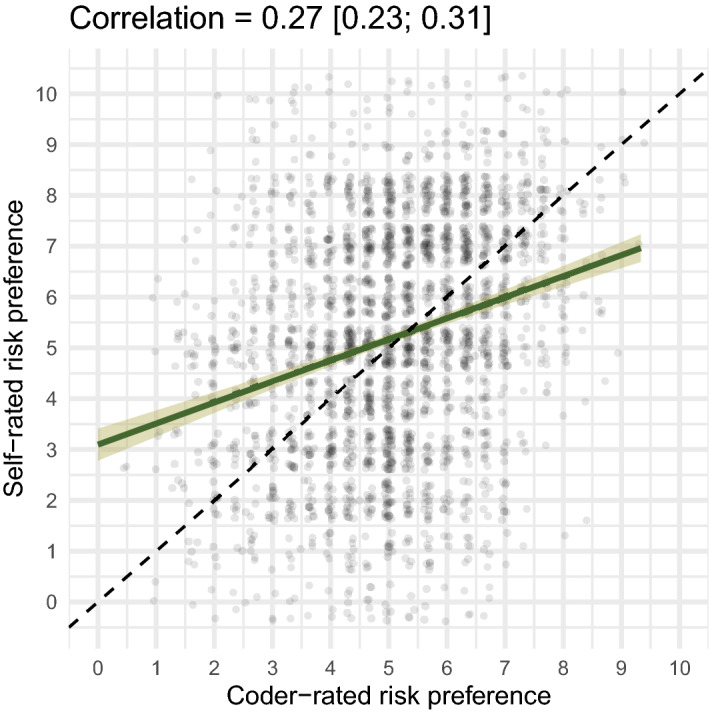
Coder accuracy. The green line shows a linear regression fit with the 95% confidence interval shaded. Along the dashed line, coder and self-ratings matched. Points were jittered slightly to reduce overplotting.

We found that coders could—based solely on the texts—estimate the stated risk preference (on a scale from 0 to 10) of the text's author by using cues such as the number of risks, whether risks were seen as worthwhile, or whether risks were avoided (see Supplement S9.8). The zero-order correlation between stated preferences and mean coder estimates was 0.27 (95% CI [0.23; 0.31], Spearman rank-correlation = 0.27) and could be described by a linear function (see Fig. 6 and Supplement S9.3). Coders agreed not only with the respondents, but also with one another: When weighted by the coders’ confidence, the intraclass correlation (ICC) was 0.63 (unweighted ICC 0.43), showing substantial agreement across coders. When coders were more confident, their judgments were also more accurate (see Supplement S9.5). Coders only minimally underestimated respondents’ risk preferences on average and less so when coders were confident (by 0.14 points, see Supplement S9.2). Coders tended towards the mean, overestimating low preferences for risk and underestimating high preferences. This tendency was more pronounced when coders were less confident in their judgment.

We carried out a social judgment analysis^50,51^ to determine which cues coders used to infer stated risk preferences and how well these cues could predict respondents’ stated preferences. Results showed that coders generally used valid cues (i.e., cues such as the number of risks which predicted both coder judgments and respondents’ stated preferences; *r* = 0.74 between predicted judgments and predicted outcomes). However, coders also used some invalid cues. For instance, coders rated those who responded vaguely as lower in risk preference, even though vagueness was not predictive of stated risk preference (see Supplement S9.8.3). A pastiche (to preserve anonymity) of a text that received the lowest rating would be: “I always keep my head out of things, and only take out loans with fixed interest rates. In the last year, I tried a new restaurant”. A pastiche for someone who received the highest rating would be “I thought about races on the motorway, and cheating on my partner. In the last year, I travelled abroad without any money”.

We also tested whether the coders could infer risk preferences from the texts equally well for respondents with different ages and genders to see whether idiosyncrasies in risk perception across age groups and gender might decrease the validity of stated preferences. We jointly tested several potential modulators of coders’ ability to infer risk preferences—study, respondent’s age, respondent’s gender, and the coder being of the same gender as the respondent—to separate their contributions to accuracy while adjusting for the number of characters written. This model was necessary due to variations between the two studies; for example, BASE-II respondents wrote more characters and were older on average than were SOEP-IS respondents. In this model, accuracy did not differ depending on the respondents’ age, gender, or the coder’s gender being the same as the respondent’s. However, BASE-II respondents were rated more accurately (i.e., coders’ evaluations matched respondents’ self-evaluations) by coders (*r* = 0.33 vs. *r* = 0.21 in SOEP-IS; see also Table 2 and Supplement S9.4), fitting the finding that considering risks worthwhile (this question was not asked in SOEP-IS) was a valid cue in the social judgment analysis. When we used multiple imputation to include respondents who did not respond or produced too little text to be rated, the association was not attenuated (*r* = 0.30 [0.26; 0.33], see Supplement S9.7). When we restricted the ratings to cases where only the first question, which focused on explaining the stated preference, was answered, the association was smaller (*r*s between 0.18 and 0.10); however, this might also be because this set of respondents produced very little text in response to the first question (Supplement S9.6).

**Table 2:** Results from a distributional regression.

Predictor	Estimates	CI (95%)
Intercept	4.27	3.66; 4.89
Stated risk preference	0.15	0.13; 0.18
σ-intercept	0.23	− 0.07; 0.51
σ-BASE-II participant	− 0.08	− 0.13; − 0.03
σ-male gender	− 0.01	− 0.05; 0.03
σ-coder has same gender	− 0.01	− 0.06; 0.03
σ-age (in decades)	0.00	− 0.01; 0.02
σ-log10 (nr. of characters)	0.05	0.03; 0.08
sd (respondent-intercept)	1.06	1.02; 1.11
sd (coder-intercept)	0.80	0.46; 1.45
sd (σ-intercept)	0.42	0.24; 0.76

The model was fit in brms^52^. We let respondents’ stated risk preferences predict the coder ratings of risk preference and let several moderators jointly predict the error term (σ) in order to disentangle their contributions. BASE-II participants were rated more accurately, when adjusting for the effects of age, gender, coder gender, and number of written characters. The model includes 2,293 respondents rated 6,863 times by nine coders (~ 3 ratings per respondent).

## Discussion

To investigate how stated preferences can be valid, we asked respondents to explain their answers to a general question about their risk preferences (GRQ)^6^. Our results show that people establish a common reference frame by seeing what preferences are revealed in the risks they themselves took, avoided, and regretted. We argue that this self-judgment taps into the general human ability for social judgment^30,33^. People constantly judge others—for instance, to quickly assess whether someone will be a steadfast ally or an unpredictable enemy^34^. One indication that self-judgments have informational value is that with just a brief glimpse into our respondents’ self-perceptions, our coders were able to infer their stated risk preferences to a significant extent. Coders did even better when, as in the BASE-II study, they had access to information about respondents’ experiences of regret. We argue that self-judgments of risk preferences take into account not just actions, but also situational constraints and internal states such as experiences of regret, or need.

The risks people thought about were highly heterogeneous. However, most respondents focused on voluntary behaviours and decisions with risk of easily observable harm, including physical, financial, and social risk. Major life decisions, especially risks taken in relationships, investments, and careers were often mentioned. Cumulative and delayed risks of harm, such as smoking or unprotected sex, were mentioned only infrequently. Furthermore, passively tolerated sources of risk from technology or natural hazards were rarely mentioned. It seems that when people consider which actions reveal their risk preferences, they think of more diverse actions than the ones experimental economists and psychologists use in the laboratory. Gambling, the most common laboratory measure of risk preferences, was mentioned only rarely, and unlike more commonly mentioned risks it was avoided and regretted more often. Seen through the eyes of our respondents, gambling is an odd risk: The precisely defined risk (in terms of probability and outcomes), the possibility of avoiding gambling entirely, and the frequency of regret all make gambling different from the more commonly mentioned risks taken in relationships, health, and careers—although investments, which were commonly mentioned, may involve a gambling element for some respondents. In contrast to the frequently employed lotteries in psychological and economic laboratories, the widely used DOSPERT questionnaire^53^ asks about a list of hypothetical behaviours that appear to better capture the full diversity of risks people can face, in terms of both risk domains and size of stakes. The DOSPERT questionnaire includes everyday behaviours such as not wearing a seatbelt, rarer behaviours like having an affair, and rare but important events like choosing a more enjoyable but less secure career. In our data, relationship and career risks were also prominent, especially among the biggest risks faced in the previous year (see also Supplement S2). These risk domains are amongst those highest on the Unknown factor of Slovic’s^47^ psychometric approach to risk perception: Decisions about whether to marry, divorce, move, quit a job, or study a particular subject are highly uncertain and can seriously alter a life’s trajectory. Respondents realised this and frequently mentioned decisions with very high stakes—which may reveal more about their own risk preferences than do the typical risks with low stakes found in the laboratory. It is possible that preferences were not only revealed through these decisions but also shaped by their consequences: as people learn through trial and error, their preferences mature^54^.

The difficulty of constructing revealed risk preference measures in domains like relationships makes representative designs, which capture the ecology of risks, less likely in the laboratory^4,50^. Much research operates under the assumption that it is possible to extrapolate from small to large risks^4,13^—that the person who gambles in a laboratory lottery will also gamble with their life and happiness. However, this assumption may not hold. We know that people are more risk averse on average when facing higher financial stakes^14,55^, but what do we know about how interindividual rank order changes when the stakes are raised? More work needs to be done to account for mounting evidence of the low criterion validity of revealed risk preference tasks^5,56^ and recent work finding that hypothetical lotteries are workable proxies of incentivised ones^57^. Any shared validity between hypothetical (or low-stakes) lotteries and stated preferences may result from a common process: People look to their past actions and experiences to construct a response to an abstract decision^22,58,59^. This general cognitive process may also explain the validity of the DOSPERT questionnaire, in which all behaviours are hypothetical and people only predict their own behaviour. Even the 30–40 items of the DOSPERT questionnaire cannot capture all the idiosyncratic yet pertinent risks our respondents listed (e.g., “buying a horse and never telling your partner”), but people could draw on idiosyncratic experiences to reasonably predict their own behaviour in standardised hypothetical situations. It is conceivable that the DOSPERT questionnaire also bolsters dialectical bootstrapping^60^, helping people come up with several responses that reflect their true preference plus noise, which can then be averaged for increased reliability (see also Supplement S3).

Because our coders could, to a significant extent, infer respondents’ risk preferences from the texts, we know the texts contained valid cues, such as the number of risks and whether risks were avoided or regretted. In fact, the correspondence between coder ratings and stated preferences (*r* = 0.27) was similar to the correspondence between risk perceptions in self-ratings and ratings by close informants (*r*s = 0.25, − 0.46^61^) and the correspondence for decisions between lotteries (*r* = 0.31) between two household members^62^. It was also close to the agreement between self and other ratings among Facebook friends for personality traits^63^. Despite their brevity—texts contained a median of ten words—the texts held pertinent information. Our social judgment analysis showed that coders relied on cues such as regret, the number of risks listed for the last 12 months, and risk avoidance. They also took note of specific risky activities, such as motorcycling and sports, and correctly inferred that respondents who listed investments as a risky activity had stated lower risk preferences.

The topics respondents thought about differed by age and gender. For example, an elderly respondent listed “getting into the bathtub” as a risk, which most younger respondents would not consider a threat. More generally, older respondents were more likely to mention risks in health and traffic, and less likely to focus on their career or gambling. Gender and age differences in risk perception and conception (i.e., focusing on favourable or unfavourable outcomes^64^) might raise doubt that there is a common denominator that allows for comparing stated risk preferences across age groups and genders. We suggest the opposite: Risk perception and conception are cues to people’s risk preference too^64,65^. In initial support of this notion, our coders—aged between 23 and 36—were equally accurate when inferring the preference of older respondents or those of the opposite gender. Given that people can agree on perceptions of risk^47,65^, as we found in our online rating study, they can also agree on what taking specific risks implies for a person’s risk preferences. Regarding the measurement of stated preferences, this interpretation leads to a more optimistic conclusion than does the widespread idea that people always anchor themselves to a social reference group (which would change according to age, location, and time). Indeed, only a minority of our respondents said they used social comparison; most said they simply thought about their past experiences and behaviours. This result may explain why, in apparent conflict with a cognitive model of personality judgments^66^, specifying reference groups reduced predictive validity in a study of conscientiousness^43^. If most people do not naturally tend to compare themselves to a reference group, they may fare worse when asked to do so. Much of the literature has focused on finding out whether questions could be improved, by specifying their frame of reference^43,44^, reference groups^66,67^, examples^42^, or specific behaviours^68,69^, or by generally reducing temporary, fluctuating influences^28,29^. In risk preference research, Blais and Weber^53^ attempted to remove any part played by differences in risk perception. Counterintuitively, leaving self-report questions fairly broad and vague may sometimes improve validity, as long as people understand the question and can draw on relevant experiences. A comprehensive single item may allow people to use their ability of social perception, and by doing so, to draw on their most pertinent and diagnostic information.

### Limitations

In order to sample responses from a cross-section of German society, we took advantage of two large longitudinal studies. The decision to use longitudinal studies implied trade-offs, especially with respect to the depth with which participants could be probed. Continued participation in longitudinal studies is important; questions and probes must therefore be brief. Future research should further develop the present closed-form questions to describe reference frames in more detail, ask about risk magnitudes, and distinguish between other-regarding and self-regarding, as well as private and public decisions. Furthermore, rewarding respondents to produce more text in response to open prompts (including possibly recording verbal answers rather than requiring typing) should help to reveal the processes behind such self-judgments (including the reasons for nonresponse). An initial study that used an elaborate process tracing method to understand stated preferences could explain the majority of the variance in self reports^24^. Hence, it seems plausible that recovering more information about the reasoning behind a stated preference would also boost rater accuracy. An analysis of those cases in which people did not respond revealed that risk averse people were more likely to respond minimally (Supplement S5). With the benefit of hindsight, it is understandable that these respondents produced, on average, much less text: it may be more difficult to remember and retrieve instances of risks they had avoided (e.g., taking a cab instead of public transportation at night) than instances of risks they had taken (e.g., traveling alone in a foreign country). If there is indeed such a mnemonic asymmetry (as is suggested by the frequent report of risks that risk averse people took voluntarily), then instructions must be designed in a way that encourages people to also access the many occasions in which they avoided specific risks. This may also increase the text production of respondents who judge themselves as more risk averse. Furthermore, revised instruction could also emphasize risks that people passively tolerate rather than actively take and risks that they take on behalf of others.

Our coders received a fixed sum, irrespective of their performance. The substantial agreement between coders and the moderate accuracy based on brief (sometimes very brief) texts give us reason to be cautiously confident in the quality of their codings. Still, one should not interpret the accuracy as estimated here on the basis of a single item as representative of the best possible performance. Our small sample of nine coders also does not shed much light onto potential heterogeneity in accuracy. Some coders may be much better than others at reading other people. Also, some of the less commonly coded categories showed subpar agreement between coders. There is no question that our ad-hoc coding scheme can be improved in these respects, especially for rarer and more ambiguous risks.

Finally, our investigation was not designed to contribute to the ongoing analyses and systematic comparisons between between stated and revealed preference measures^5^. Yet, our conceptual approach—elaborating the process of self-perception according to which people come to “know” their preferences and internal states through memory samples of their own relevant behaviours—may also be a fruitful framework for finding the extent to which similar inferential processes play a role in producing behaviours in revealed preference tasks.

## Conclusion

What many researchers feel is a weakness of stated preferences (“cheap talk”) might actually be a strength^15^. The fairly vague, almost projective nature of a comprehensive single-item question allows people to refer back to their diagnostic memories and behaviours using a well-honed human capacity for social perception. People with different risk perceptions and conceptions could be problematic for the intersubjective comparability of their answers^64^, but we find that people (our coders) can generally agree on what risky behaviours imply for a person’s risk preference, irrespective of age and gender. The shared social perception of risks fosters agreement and comparability, as well as the validity of risk preferences. This does not imply that self-reports are always suitable. For instance, applicants for a position as a financial manager could foil an attempt to screen for risk-seekers by simply dissembling—just as they could in typical laboratory tasks, where stakes are generally low.

Far from “cheap talk,” self- and informant-reports are based on informative and diagnostic cues and permit people to apply the full might of social perception to themselves, enabling intersubjective agreement. These results suggest that researchers in economics and psychology can learn from the experts on person perception: their study participants. By inferring risk preferences from diagnostic behaviours and experiences, people essentially adopt the logic of the revealed preference approach—namely, that otherwise unobservable preferences reveal themselves in behaviour. Ironically, the revealed preference approach appears to have found new significance in research on stated risk preferences.

## Materials and methods

All questions and materials needed to reproduce the study have been shared on Open Science Framework (OSF) at osf.io/eun4r/. The main questions can be found in Supplement S4. The stated preferences were collected in the 2017 interim wave of the Berlin Aging Study II (BASE-II^45^) and the 2017/2018 wave of the SOEP Innovation Sample (SOEP-IS^46^). Both studies are age-heterogeneous longitudinal panel studies. SOEP-IS aims to representatively sample private households in Germany; BASE-II is a convenience sample of younger and older adults from Berlin, Germany. Participants in both studies had already answered the general and domain-specific risk questions in previous waves. In the 2017/2018 wave, 3,493 respondents answered the GRQ and 3,089 answered several questions that elicited free-text source reports. Both studies have been documented on https://paneldata.org. Fieldwork for SOEP-IS started in September 2017 and ended in February 2018. Questionnaires for BASE-II were mailed out at the beginning of November 2017; data collection ended in January 2018. The online rater sample was recruited from online panels psytests.de and psyweb.uni-muenster.de from April to August 2018. Participants could win one of 50 Amazon coupons worth €25 each in a lottery. The coders were recruited from the participant pool of the Max Planck Institute for Human Development and were paid €180 each. Descriptive statistics for all samples are summarised in Table 3. The anonymised data for the online rating study is available on OSF. The SOEP-IS data can be obtained from the SOEP re-analysis archive; the BASE-II data can be obtained from the BASE-II Steering Committee. All participants provided their written informed consent. The SOEP study was approved by the Institutional Review Board of the SOEP. The BASE-II study was approved by the Ethics Committees of the Max Planck Institute for Human Development and Charité-Universitätsmedizin Berlin. The online rating and the coding study were approved by the Institutional Review Board of the Max Planck Institute for Human Development. The studies were performed in accordance with all relevant guidelines and regulations.

**Table 3:** Demographic statistics for the three samples.

	SOEP-IS (n = 1,928)		BASE-II (n = 1,569)		Online raters (n = 944)		Coders (n = 9)
	Mean (SD)	Missing	Mean (SD)	Missing	Mean (SD)	Missing	Mean (SD)
Age	53.4 (18.6)	0	66.6 (15.9)	0	46.8 (17.6)	272	27.9 (4.4)
Male	47%	0	48%	0	39%	281	56%
General risk Q	4.6 (2.4)	0	5.2 (2.3)	4	4.4 (2.1)	123	
No. of words	7.5 (8.0)	274	18.0 (15.5)	138			
Text length	51 (51)	274	135 (106)	134			
Codeable topics Q1	46%	0	80%	0			
Codeable topics Q2	40%	0	67%	0			

There were no missing values for the coders. A subsample of n = 825 online raters rated the individual hazards (n = 119 ended the study before the ratings).

*SD* standard deviation.

### Measures

#### Stated preferences

Stated preferences were measured using the GRQ^6^. After respondents answered this question, they were asked a series of follow-up questions. We slightly reduced the number of questions in SOEP-IS compared to BASE-II to fit the time requirements of the panel study. In both studies, the first follow-up question was “Which events, behaviour, or persons did you think about when you indicated a number for your risk preference?” Participants could check multiple options: “own experiences,” “own behaviour,” “my behaviour compared to others,” “the consequences of my behaviour for me,” “the consequences of my behaviour for others,” and “what people around me say about my risk preference.” In SOEP-IS, respondents could also choose from several nonresponse options: “gave my answer spontaneously without deliberating a great deal,” “none of these,” and “no answer.” In BASE-II, a second multiple choice question asked respondents whether they thought about one or more of the following options: “how I presently behave in my day-to-day life,” “how I behaved in the past,” “how I will behave in the future,” “how prepared for risks I would like to be,” and “did not think about myself.” In both studies, the closed-form questions were followed by two free-text questions: “Which concrete experiences or behaviours—yours or others’—did you think about? Please give keywords” and “In which situations in the last 12 months were you prepared to take risks? List up to three situations in which you took the biggest risks. Keywords suffice.” In BASE-II only, respondents were then asked, “And were the risks worth it?” The free-text questions were designed to be maximally open-ended and to encourage respondents to give detailed answers, suitable for coding, through a conversational style. The closed-form questions were designed to additionally elicit information on reference frames that participants were unlikely to mention themselves.

The BASE-II respondents filled out paper-and-pencil questionnaires and returned them by mail. They were given four lines to write on for each free-text question. Their responses were later transcribed by student assistants. In SOEP-IS, respondents answered verbally and the interviewer transcribed their answers during computer-assisted personal interviewing. BASE-II respondents gave valid and elaborate answers to the free-text questions more frequently than did the SOEP-IS participants: 92%, compared to 86% (*n*s = 1,435; 1,654), answered at least one of two free-text questions. BASE-II respondents wrote a median of 106 characters; the median for SOEP-IS respondents was 35 characters. Texts by BASE-II respondents were sufficiently informative to code risk topics for 1,248 responses to the question asking them to explain their thinking for the stated preferences and for 1,056 responses to the question asking about risks taken in the last year. Given the shorter responses in SOEP-IS, topics were codeable only for *n*s = 890/773 free-text responses (see also Supplement S5).

#### Text coding

The texts written by the BASE-II and SOEP-IS participants were hand-coded by a set of nine coders (aged 23–36, four women) over several days. We randomly divided the full-text answers into two sets of 1,000 and one set of 1,059 answers. The coding scheme was derived through a mixture of a deductive approach (hazards listed in the literature^47^) and an inductive approach (further hazards mentioned in the texts). For initial training, all coders coded a set of the same 50 texts. Afterwards, the coding scheme was refined and agreement was checked according to Fleiss’ kappa. Points of disagreement about the scheme between coders were resolved by the first author (RCA). For the remainder of the texts, three coders coded each text. Coders tended to agree on the presence of risk domains; Fleiss’ kappas were above 0.70 for all coder groups (see Supplement 9.8.1) and all risks except safety and crime (κ ≥ 0.49, because coders did not always agree whether respondents were perpetrators or victims of crime), and cataclysms (κ = 0.00–0.61, but this category was very rare). They also noted whether the texts mentioned risks that were taken or avoided (here, agreement was only slight: κ = 0.04–0.18) as well as whether respondents thought the risk had been worthwhile (κ = 0.71–0.77).

Coders saw all the answers to the free-text questions given by a respondent simultaneously in case the answers referenced each other. They did not see the answers to the closed-form questions or other identifying characteristics. First, coders judged whether meaningful topics or situations were mentioned in the response. If not, they could code whether the response was gibberish, a statement of absence, or similar. They then coded the presence of the topics from the coding scheme (e.g., health, relationships) for each of the two free-text questions. Some risk domains included more specific hazards as subcategories (e.g., health: surgery or relationships: divorce) that could be coded (see Supplement S4.2). For the first question, which asked respondents to explain their thinking for their stated preferences, coders noted whether the situations and events described focused on risk prevention or promotion (the second question was explicitly about risks taken in the last year and therefore could not be codified this way). For the question asking whether risks were worthwhile, which appeared only in BASE-II, coders noted whether the respondents thought the risk had been worthwhile or whether they were unable to tell so far (e.g., long-term financial risks). Finally, the coders rated the respondents on their answer to the GRQ. For our analyses, we chose the consensus value given by the coders (i.e., the coding by at least two coders) or the mean for continuous values. For the 50 texts that we used to train coders, we omitted the data from the first six coders before aggregation to keep the procedure comparable for all texts.

### Analyses

Our data processing code, statistical analyses, and detailed results are reproducibly documented on OSF (osf.io/eun4r/).

#### Online rating of risk perceptions

Online participants rated the hazards from our coding scheme (e.g., moving in together, smoking) on 22 characteristics (e.g., observability, reducibility). The online raters did not read the free texts; instead, each rater rated three to five randomly drawn hazards on all characteristics. To measure the reliability of the average ratings, we computed average ICCs for each characteristic for an average of 17 aggregated ratings, which was the lowest number of ratings any individual hazard had received (median = 37). Average ICCs ranged from 0.73 (whether risks were known to science) to 0.97 (whether risks were related to social position). These ICCs are lower bounds, as most risks were rated by more than 17 raters (see Supplement S8.1 for all ICCs). Because it is not possible to meaningfully answer questions such as “Are health risks known to science?” the online sample did not rate broad and vague risk domains such as health and traffic; instead, we averaged the ratings of the constituent hazards to arrive at values for the risk domains. To construct a familiar map of the risk domains and hazards for our readers, we extracted the factors Dread and Unknown according to a confirmatory specification based on 16 characteristics from Slovic^47^. We could approximately replicate the coordinate system positions of risks in Slovic^47^, fulfilling our limited aim, but—probably because we had added nonmortality, social risks—fit indices fell short (see Supplement S8.2). Owing to a programming error, the hazards “gambling,” “travel,” and “surgery” were not rated by the online sample and are therefore not shown in Fig. 2.

#### Coder-estimated risk preferences

Coders had indicated whether the text contained direct hints to the authors’ gender, age, or place of residence, such as, “My husband lost at bingo in our retirement home in Munich.” Because such hints might serve as cues to the stated risk preference, given age and gender differences in risk preferences, but would be unrelated to risk conceptions per se, we restricted the main analysis to the majority (97%, n = 2,310) of texts which contained no direct hints. Even indirect hints, such as considering “getting into the bathtub” a risk, seemed to play little role: accuracy was not attenuated when we adjusted for respondent age and gender (see Supplement S9.1).

Coders could tell when they had usable information. Accuracy was r = 0.06 when coders said they were guessing, but r = 0.45 when they had maximal confidence (see Supplement S9.5). Coders did not learn to judge more accurately with practice; we had expected this since they received no feedback.

## Supplementary information

Supplementary Information.
