# Supplementary material for: How people know their risk preference
Source: Sci Rep. 2020 Sep 21;10:15365. doi: 10.1038/s41598-020-72077-5 (PMC7505965; doi:10.1038/s41598-020-72077-5)
Supplement: Supplementary file 1 — Supplementary Information. [file 41598_2020_72077_MOESM1_ESM.pdf]

Supplement: How people know their risk preference

Arslan, R. C., Brümmer, M., Drewelies, J., Hertwig, R., & Wagner, G. G.

Contents

S1 Criterion validity of stated and revealed preferences **2**

S2 The gap between domain-specific and global items **4**

S3 General single items versus multiple specific items **5**
S3.1 Comparison between General Risk Question and DOSPERT questionnaire 5

S4 Questions and coding scheme **7**
S4.1 Respondent sample 7
S4.2 Coding scheme 9
S4.3 Rating of risk categories 16

S5 Nonresponse analysis **20**

S6 Reference frames **22**
S6.1 By age 26
S6.2 By gender 27

S7 Topics **28**
S7.1 Reported topics by question 28
S7.2 Combinations 29
S7.3 Detail level 30
S7.4 Age trends 31
S7.5 Multiple imputation in case of nonresponse 33
S7.6 Gender differences 35
S7.7 Word clouds 36

S8 Quantifying risks according to psychometric characteristics **40**
S8.1 Agreement across raters 40
S8.2 Confirmatory factor analysis 42
S8.3 Rated item means 44

S9 Can coders predict risk preference from the text? **45**
S9.1 Unmasking 45
S9.2 Rank-order and mean differences 46
S9.3 Linearity 48
S9.4 Differences by study 49
S9.5 Calibration 50
S9.6 Only first question 51
S9.7 Multiple imputation in case of nonresponse 52
S9.8 Cues 53

S10 Supplementary References **61**

S1 Criterion validity of stated and revealed preferences

Over the years, several teams have investigated associations between revealed preference tasks and stated preferences, estimating both intercorrelations across measures and retest stability (Charness et al., 2020; Coppola, 2014; Frey et al., 2017; Lönnqvist et al., 2015; Pedroni et al., 2017; Tynan, 2018). A comparative study of retest stability (Frey et al., 2017) found higher stability for several measures of stated preferences than for most measures of revealed preferences. For a review of older retest stability research, see Chuang and Schechter (2015).

A consistent finding in the literature is that retest stabilities for experimental/revealed preference type measures of risk preferences are low, even over short intervals, and lower than the stability of stated preferences. Most studies found fairly low convergence between stated and revealed preferences, although there is heterogeneity in the literature with estimated relationships varying from 0 to 0.5 (Pearson correlations).

Several teams have reviewed the comparative studies of different measures of risk preferences (Bran & Vaidis, 2019; Charness et al., 2013; Galizzi et al., 2016; Harrison et al., 2005; Hertwig et al., 2019; Mata et al., 2018). Some have also conducted head-to-head comparisons of the criterion validity (sometimes termed "predictive validity" and/or "generalizability") of revealed and stated preferences–that is their ability to predict behaviours of interest in the real world. These have included behaviours such as buying stocks, being self-employed, taking health risks such as smoking, sexual risk taking, and savings. We think the criterion validity of measures is particularly interesting, because it can speak more directly to the question of whether research findings will generalize to the real world than findings of reliability and stability can. Since the literature is disconnected across economics and psychology, we summarise key findings in Table S1.

Table S1: Summary of the criterion validity findings in the literature

Study	Summary of criterion validity results	N
Szrek et al., 2012	SOEP-GRQ and DOSPERT predicted health risks (smoking, problem drinking, seat belt non-use, and risky sexual behaviour) better than HL and BART did.	351
Tynan, 2018	DOSPERT but not lab risk tasks (BART, Iowa Gambling, Columbia Card Sorting) predicted RISQ (self-reported risky behaviours).	383
Coppola, 2014	Specific SOEP questions and DOSPERT predicted risks (smoking, self-employment, risky assets, sports, private disability insurance) better than hypothetical lotteries did.	1,302
Falk et al., 2018	A combined index of SOEP-GRQ and a hypothetical lottery predicted various risky behaviours (e.g., savings) within and across countries.	80,337
Frey et al., 2017	SOEP-GRQ, DOSPERT and other stated preferences predicted self-reported propensity measures (drinking, smoking, gambling, drug abuse, aggressive behaviour, sexual risks, risks at work, risky behaviours in past 12 months) better than various task measures (including lotteries, BART) did.	1,507
Galizzi et al., 2016	Limited criterion validity for the criteria smoking, junk food consumption, fruit and vegetable consumption, body mass index (BMI), and heavy drinking for revealed and stated preference measures. SOEP-GRQ and SOEP-Finance predicted savings and heavy drinking better than incentivised lotteries, whereas lotteries better predicted BMI and fruit and vegetable consumption.	T1:661/T2:413
Beauchamp et al., 2017	In the male subsample, SOEP-GRQ and two hypothetical gambling tasks predicted investment decisions, self-employment, drinking, and smoking Only the SOEP-GRQ predicted all of these significantly, but the hypothetical gambles explained more variation in investment decisions.	11,418
Charness et al., 2020	Neither the SOEP-GRQ, nor several revealed preference tasks significantly predicted savings, risky investments, insurance, deductibles, self-employment, or owning real estate, but statistical power was generally quite low.	86-234
Dohmen et al., 2011	The SOEP-GRQ and domain-specific SOEP questions predicted self-employment, smoking, owning stocks, and being active in sports. A hypothetical lottery significantly predicts only owning stocks (in a smaller subsample).	7,345-13,571

Note:
The table is not based on a systematic literature search; instead, it aims to highlight a few of the most important studies that compared stated and revealed preferences measures head-to-head. We did not include studies with fewer than 200 participants and only included outcomes that indexed real-life behaviour outside the laboratory (no economic games and incentivised tasks). Charness et al. (2020) had sample sizes below our cutoff for some outcomes but not others.
SOEP-GRQ: The General Risk Question we used in this study.
DOSPERT: Domain-Specific Risk-Taking Scale
BART: Balloon Analogue Risk Task

S2 The gap between domain-specific and global items

In the SOEP and BASE-II studies, participants also answered single items about domain-specific risk attitudes (driving, finances, sports, career, health, and trusting others). All of them have a lower mean than the general risk preference item. How can the risk preference across risk domains be higher than its constituent parts? One possible explanation is an inconsistent response behaviour. Another is risks that matter to people are not queried in the domain-specific items, such as relationships. If people perceive themselves as taking many risks in this area, it could explain the gap left in comparison with the General Risk Question's mean. The only item related to relationships is about trusting strangers, which taps into just one small aspect of risk in relationships.

Table S2: General and domain-specific risk preferences

variable	mean	general	car	finance	sports	job	health	trust
general	4.8	1.00	0.40	0.44	0.47	0.48	0.37	0.29
car	3.1	0.40	1.00	0.40	0.44	0.36	0.40	0.20
finance	2.2	0.44	0.40	1.00	0.35	0.35	0.34	0.27
sports	4.0	0.47	0.44	0.35	1.00	0.51	0.47	0.29
job	4.1	0.48	0.36	0.35	0.51	1.00	0.41	0.25
health	3.2	0.37	0.40	0.34	0.47	0.41	1.00	0.27
trust	4.0	0.29	0.20	0.27	0.29	0.25	0.27	1.00

Note:
Shows the means of each item and the intercorrelations.

S3 General single items versus multiple specific items

Given that a general factor of risk preference seems to explains a large portion of the responses to the DOSPERT questionnaire, it may be an uneconomical solution for studies aiming to measure general risk preference (Highhouse, Nye, & Zhang, 2017). Survey methodologists and psychometricians (Revelle et al., 2016) have long recommended that when time is short, researchers should randomly ask a few questions from a question pool to each participant instead of reducing survey length by using the same few items for everyone, thereby sacrificing construct breadth. However, this recommendation is rarely implemented, probably mainly because researchers feel it is inconvenient to implement and analyse. Given the well-known result that specific scales predict specific criteria best and broad scales are best at predicting broad criteria (Highhouse et al., 2017; Mõttus, Bates, Condon, Mroczek, & Revelle, 2017; Mõttus, Kandler, Bleidorn, Riemann, & McCrae, 2017), we note that the SOEP General Risk Question exhibited criterion validity for risks such as smoking, drinking, and gambling (Frey et al., 2017), even though respondents rarely mentioned these behaviours in our study and instead focused on high-stakes risks in finance, relationships, career, and traffic. Future research should test whether comprehensive single questions could preserve construct breadth when asking random specific questions from a bigger pool is inconvenient.

S3.1 Comparison between General Risk Question and DOSPERT questionnaire

We reanalysed data (https://osf.io/tckbj) from the Basel—Berlin Risk Study (Frey et al., 2017; Pedroni et al., 2017) to compare the approach taken in the General Risk Question (GRQ) with that in the DOSPERT questionnaire. The GRQ is a fairly open-ended question that allows participants on real experiences or anything else they deem relevant, while the DOSPERT questionnaire lists many concrete hypothetical risks or situations and asks participants whether they would take a risk in that situation.

We took the propensity measures—that is concrete questions on real-world risk taking—as criteria and contrasted the correlation between them and the GRQ with the correlation between the propensity measures and all of the DOSPERT items.

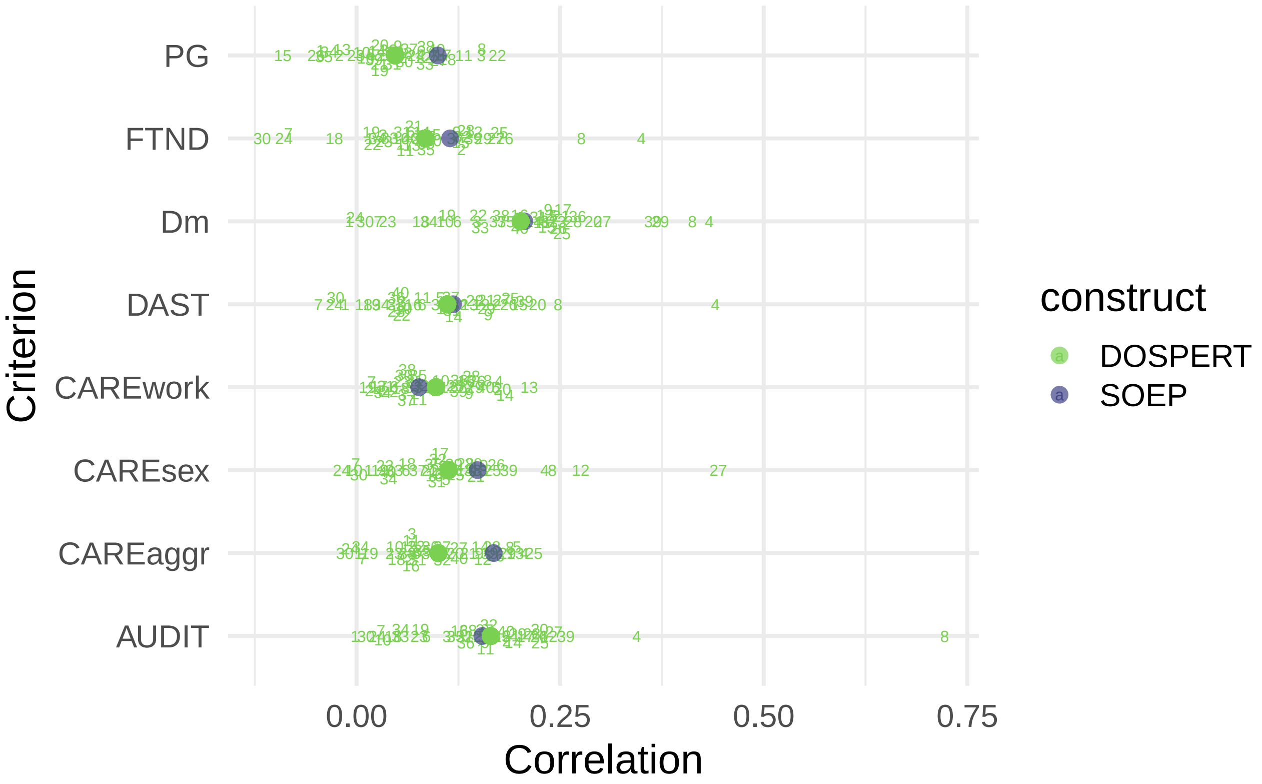

Figure S1: The General Risk Question consistently predicted the propensity measures in the Berlin-Basel Risk Study. Its correlation with the propensity measures was close to the average DOSPERT item.
AUDIT: Alcohol use disorders identification test.
FTND: Fagerström test for nicotine dependence.
PG: Pathological gambling.
DAST: Drug Abuse Screening Test.
CAREaggr: Risky situations, aggressive behaviour.
CAREsex: Risky situations, sexual behaviour.
CAREwork: Risky situations, behaviour at work.
Dm: Risky behaviours in the past month.

We averaged the correlations between each DOSPERT item and each propensity variable and between the SOEP-GRQ and each propensity variable. We then subtracted the averaged correlation for each DOSPERT item from that for the SOEP-GRQ. The SOEP-GRQ explained about as much as any single DOSPERT item on average (average r difference: 0.02, range: -0.19;0.13).

We also used the same procedure, but sampled seven random items from the 40 DOSPERT items 1,000 times. We then compared their correlations with the propensity variables with the correlation of a general factor extracted from the seven SOEP risk questions (the GRQ and six domain-specific questions).

The SOEP items explained about as much as did any random subset of seven DOSPERT items on average (average r difference: 0, range: -0.14;0.11).

S4 Questions and coding scheme

S4.1 Respondent sample

The following questions were posed to respondents.

S4.1.1 General Risk Question (in BASE-II and SOEP-IS)

Wie schätzen Sie sich persönlich ein: Versuchen Sie im allgemeinen, Risiken zu vermeiden oder sind Sie im allgemeinen ein risikobereiter Mensch? [How do you assess yourself: Do you generally try to avoid risks or are you generally prepared to take risks?] (rated on scale from 0 to 10)

S4.1.2 Social/experiential reference frame (in BASE-II and SOEP-IS)

An welche Ereignisse, Verhaltensweisen oder Personen haben Sie gedacht, als Sie die Zahl für Ihre Risikobereitschaft angegeben haben? (Mehrfachantwort möglich) [Which events, behaviours, or people did you think about, when you indicated a number for your risk preference? (multiple options can be checked)]

- Eigene Erlebnisse [own experiences]
- Eigenes Verhalten [own behaviour]
- Mein Verhalten im Vergleich mit dem Verhalten anderer Personen [My behaviour compared to the behaviour of others]
- An die Folgen meines Verhaltens für mich [about the consequences of my behaviour for me]
- An die Folgen meines Verhaltens für andere [about the consequences of my behaviour for others]
- Habe daran gedacht, was mein Umfeld mir über meine Risikobereitschaft sagt [thought about what people around me say about my risk preference]
- Habe die Angabe ganz spontan ohne großes Überlegen und Nachdenken gemacht [answered spontaneously without deliberating a great deal] (only in SOEP-IS)
- Nichts davon [none of these] (only in SOEP-IS)
- Keine Angabe [no answer] (only in SOEP-IS)

S4.1.3 Temporal reference frame (only in BASE-II)

Und als Sie Ihre Risikobereitschaft mit einer Zahl eingeschätzt haben: Haben Sie daran gedacht… (Mehrfachantwort möglich) [And when you assessed your risk preference with a number; did you think about… (Multiple options can be checked)]

- …wie Sie sich gegenwärtig im Alltag verhalten? [how you currently behave in your day-to-day life?]
- …wie Sie sich in der Vergangenheit verhalten haben? [how you behaved in the past?] (repeated erroneously at the end of the list)
- …wie Sie sich in der Zukunft verhalten werden? [how you will behave in the future?]
- …wie risikobereit Sie gerne wären? [how prepared for risks you would like to be?]
- …habe nicht an mich gedacht [did not think about myself]

S4.1.4 Q1. Concrete events (in BASE-II and SOEP-IS)

An welche konkreten Erlebnisse oder Verhaltensweisen – egal ob von Ihnen oder anderen – haben Sie gedacht? Bitte nennen Sie Stichworte [Which concrete experiences or behaviours—yours or others'—did you think about? Please give keywords.] (Open questions with four lines to write on)

S4.1.5 Q2. Biggest risks taken in the last 12 months (in BASE-II and SOEP-IS)

In welchen Situationen waren Sie in den letzten 12 Monaten bereit, ein Risiko einzugehen? Nennen Sie bitte bis zu drei Situationen, in denen Sie am meisten Risiko eingegangen sind. Stichworte genügen. [In which situations in the last 12 months were you prepared to take risks? List up to three situations, in which you took the biggest risks. Keywords suffice.] (Open questions with four lines to write on)

S4.1.6 Worthwhile (only in BASE-II)

Und haben sich die Risiken gelohnt? [And were the risks worthwhile?] (Open questions with four lines to write on)

S4.2 Coding scheme

The following coding scheme was used by our coders (implemented as a survey in https://formr.org). Coders followed a coding guide which can be found on OSF (https://osf.io/fv7tk/, only in German).

Table S3: Coding scheme used by the coders to quantify topics and themes in the source reports

name	type	label	label_en
contains_topics_q1*	mc_button	Enthält der Text kodierbare Situationen oder Themenfelder?	Does the text contain codable situations or topics?
contains_situations_q1*	mc	Bezieht sich der Text auf...	Does the text relate to...
number_topics_q1	mc_button	Wieviele separate Situationen und/oder Themenfelder wurden genannt?	How many separate situations or topics were mentioned?
meaningful_entry_q1	mc	Was wurde eingetragen?	What was entered?
topics_q1*	mc_multiple_button	#### Kommen diese ___Überthemen___ vor?	Are these main topics present?
health_q1*	mc_multiple_button	#### Unterthemen ___Gesundheit <i class=”fa fa-3x fa-heart”></i>___	Subtopics Health
crime_q1*	mc_multiple_button	#### Unterthemen ___Gesetzesbrüche <i class=”fa fa-3x fa-user-secret”></i>___	Subtopics Crime
safety_q1*	mc_multiple_button	#### Unterthemen ___Alltag & Sicherheit <i class=”fa fa-3x fa-calendar-alt”></i>___	Subtopics Everyday Life & Safety
relationships_q1*	mc_multiple_button	#### Unterthemen ___Beziehungen <i class=”fa fa-3x fa-users”></i>___	Subtopics Relationships
traffic_q1*	mc_multiple_button	#### Unterthemen ___Verkehr <i class=”fa fa-3x fa-rocket”></i>___	Subtopics Traffic
cataclysm_q1*	mc_multiple_button	#### Unterthemen ___Katastrophen<br><i class=”fa fa-3x fa-bullhorn”></i>___	Subtopics Cataclysm
money_q1*	mc_multiple_button	#### Unterthemen ___Investitionen/Finanzen <i class=”fa fa-3x fa-money-bill-alt”></i>___	Subtopics Investments
sports_q1*	mc_multiple_button	#### Unterthemen ___Sport <i class=”fa fa-3x fa-circle”></i>___	Subtopics Sports

Table S3: Coding scheme used by the coders to quantify topics and themes in the source reports *(continued)*

<table>
<tr><th>name</th><th>type</th><th>label</th><th>label_en</th></tr>
<tr><td>risks_taken_or_not</td><td>mc</td><td>Hat die Person Risiken angegeben, die sie ___selbst eingegangen___ ist?</td><td>Did the person mention risk they took ___themselves___?</td></tr>
<tr><td>code_situations_12months</td><td>note</td><td>“‘{r} library(soeptexts) this = soeptexts[soeptexts$id == code_id,] “‘
___In welchen Situationen waren Sie in den letzten 12 Monaten bereit, ein Risiko einzugehen? ___:
> ‘r stringr::str_replace_all(this$situations_last_12_months, ”(\r\n|\n|\r)”, ”
”)‘</td><td>Text of Q2</td></tr>
<tr><td>contains_topics_q2*</td><td>mc_button</td><td>Enthält der Text kodierbare Situationen oder Themenfelder?</td><td>Does the text contain codable situations or topics?</td></tr>
<tr><td>contains_situations_q2*</td><td>mc</td><td>Enthält der Text konkrete Situationen?</td><td>Does the text contain concrete situations?</td></tr>
<tr><td>number_topics_q2</td><td>mc_button</td><td>Wieviele separate Situationen und/oder Themenfelder wurden genannt?</td><td>How many separate situations or topics were mentioned?</td></tr>
<tr><td>meaningful_entry_q2</td><td>mc</td><td>Was wurde eingetragen?</td><td>What was entered?</td></tr>
<tr><td>topics_q2*</td><td>mc_multiple_button</td><td>Kommen diese Überthemen vor?</td><td>Are these main topics present?</td></tr>
<tr><td>health_q2*</td><td>mc_multiple_button</td><td>#### Unterthemen ___Gesundheit
<i class=”fa fa-3x fa-heart”></i>___</td><td>Subtopics Health</td></tr>
<tr><td>crime_q2*</td><td>mc_multiple_button</td><td>#### Unterthemen ___Gesetzesbrüche
<i class=”fa fa-3x fa-user-secret”></i>___</td><td>Subtopics Crime</td></tr>
<tr><td>safety_q2*</td><td>mc_multiple_button</td><td>#### Unterthemen ___Alltag & Sicherheit
<i class=”fa fa-3x fa-calendar-alt”></i>___</td><td>Subtopics Everyday Life & Safety</td></tr>
<tr><td>relationships_q2*</td><td>mc_multiple_button</td><td>#### Unterthemen ___Beziehungen
<i class=”fa fa-3x fa-users”></i>___</td><td>Subtopics Relationships</td></tr>
<tr><td>traffic_q2*</td><td>mc_multiple_button</td><td>#### Unterthemen ___Verkehr
<i class=”fa fa-3x fa-rocket”></i>___</td><td>Subtopics Traffic</td></tr>
<tr><td>cataclysm_q2*</td><td>mc_multiple_button</td><td>#### Unterthemen ___Katastrophen
<i class=”fa fa-3x fa-bullhorn”></i>___</td><td>Subtopics Cataclysm</td></tr>
</table>

Table S3: Coding scheme used by the coders to quantify topics and themes in the source reports *(continued)*

name	type	label	label_en
money_q2*	mc_multiple_button	#### Unterthemen ___Investitionen/Finanzen <i class=”fa fa-3x fa-money-bill-alt”></i>___	Subtopics Investments
sports_q2*	mc_multiple_button	#### Unterthemen ___Sport <i class=”fa fa-3x fa-circle”></i>___	Subtopics Sports
risk_worth_it	note	“‘{r} library(soeptexts) this = soeptexts[soeptexts$id == code_id,] “‘ ### ___Und haben sich die Risiken gelohnt?___: > ‘r stringr::str_replace_all(this$worth_it, ”(\r\n|\n|\r)”, ”<br>”)‘	Text of Q3
risk_worth_it_coded	mc	Hat die Person angegeben, dass sich die Risiken eher gelohnt haben?	Did the person say, that the risks were worth it?
wrap_up	note	### Abschluss	Wrap-up
risk_preference_rated	rating_button	Wie beurteilen Sie die Person, die diese Antworten gegeben hat: Ist sie im Allgemeinen ein risikobereiter Mensch oder versucht sie, Risiken zu vermeiden?	How do you assess the person who gave these answers? Is it someone who is, in general, prepared to take risks, or do they try to avoid risks?
risk_preference_confidence	rating_button	Wie sicher sind Sie sich bei dieser Einschätzung?	How sure are you about your assessment?
unmasking	mc_multiple	Waren Geschlecht, Alter, Wohn- oder Aufenthaltsorte erkennbar?	Were there hints about gender, age, abode, or place names?
notes*	textarea	Haben Sie noch Anmerkungen zum Kodierprozess, die durch die obigen Fragen nicht abgedeckt wurden?	Do you notes about the coding process that were not covered above?
finish	submit	Kodieren	Code

Note:
Fields marked with * were optional

Table S4: Available choices in the coding scheme

list_name	name	label	label_en
topics	health	Gesundheit <i class=”fa fa-3x fa-heart”></i>	Health
topics	crime	Gesetzesbrüche <i class=”fa fa-3x fa-user-secret”></i>	Crime
topics	relationships	Beziehungen <i class=”fa fa-3x fa-users”></i>	Relationships
topics	safety	Alltag & Sicherheit <i class=”fa fa-3x fa-calendar-alt”></i>	Everyday Life & Safety
topics	traffic	Verkehr <i class=”fa fa-3x fa-rocket”></i>	Traffic
topics	cataclysm	Katastrophen<br><i class=”fa fa-3x fa-bullhorn”></i>	Cataclysm
topics	investments	Investitionen/Finanzen <i class=”fa fa-3x fa-money-bill-alt”></i>	Investments
topics	sports	Sport <i class=”fa fa-3x fa-circle”></i>	Sports
topics	career	Karriere/Ausbildungsentscheidungen <i class=”fa fa-3x fa-graduation-cap”></i>	Career/Education
topics	travel	Reisen <i class=”fa fa-3x fa-ship”></i>	Travel
topics	gambling	Glücksspiel, Wetten <i class=”fa fa-3x fa-dice”></i>	Gambling
topics	other	Andere	Other
health_topics	smoking	Rauchen	Smoking
health_topics	coffee	Kaffee	Coffee
health_topics	sex	Sex	Sex
health_topics	drinking	Alkoholkonsum	Alcohol consumption
health_topics	cannabis	Cannabiskonsum	Cannabis consumption
health_topics	other_drugs	Andere Drogen	Other drugs
health_topics	pesticides	Pestizide	Pesticides
health_topics	air_pollution	Luftverschmutzung	Air pollution
health_topics	medication_side_effects	Nebenwirkungen von Medizin	Medication side effects

Table S4: Available choices in the coding scheme *(continued)*

list_name	name	label	label_en
health_topics	unhealthy_food	Ungesundes Essen	Unhealthy food
health_topics	gmo_food	Genmanipuliertes Essen	GMO food
health_topics	other_toxins	Andere Giftstoffe	Other toxins
health_topics	vaccines	Sich impfen	Vaccines
health_topics	vaccine_avoidance	Sich nicht impfen	Vaccine avoidance
health_topics	other_longterm	Andere Langzeitrisiken	Other long-term risks
health_topics	operation	Operation	Surgery
health_topics	other_immediate_risks	andere, sofortige Risiken	Other immediate risks
relationship_topics	moving_professional	Umziehen (berufliche Risiken)	Moving (professional risks)
relationship_topics	moving_social	Umziehen (soziale Risiken)	Moving (social risks)
relationship_topics	moving	Umziehen (allgemein)	Moving (generally)
relationship_topics	moving_in	Zusammenziehen (mit Partner)	Moving in together (with partner)
relationship_topics	marriage	Heirat	Marriage
relationship_topics	pregnant	Schwangerschaft/Kinder kriegen (für die Schwangere)	Pregnancy/having children (for the pregnant woman)
relationship_topics	divorce	Scheidung	Divorce
relationship_topics	separation	Trennung	Separation
relationship_topics	affairs	Affäre	Affairs
relationship_topics	speaking_out	die eigene Meinung sagen	Speaking out about one’s opinion
relationship_topics	sticking_by	Zu jemand halten	Sticking by someone
relationship_topics	children	Konflikte mit den eigenen Kindern eingehen	Conflicts with own children
relationship_topics	children_general	eigene Kinder (allgemein)	Other mention of own children
relationship_topics	colleagues	Kollegen	Mention of colleagues
relationship_topics	conflicts	Konflikte (allgemein)	Conflicts (generally)
relationship_topics	other_relationship_risk	Andere	Other relationship risks
crime_topics	commit_misdemeanors	Ordnungswidrigkeit begangen	Commit misdemeanours
crime_topics	commit_crime	Verbrechen begangen	Commit crimes
crime_topics	other_crime_risk	Andere	Other crime risks
traffic_topics	car	Auto fahren <i class=”fa fa-car”></i>	Driving
traffic_topics	bicycling	Fahrrad fahren <i class=”fa fa-bicycle”></i>	Bicycling

Table S4: Available choices in the coding scheme *(continued)*

list_name	name	label	label_en
traffic_topics	motorcycle	Motorrad fahren <i class=”fa fa-motorcycle”></i>	Motorbiking
traffic_topics	flying	Fliegen <i class=”fa fa-plane”></i>	Flying
traffic_topics	bus	Bus, Tram, U-Bahn <i class=”fa fa-bus”></i> <i class=”fa fa-subway”></i>	Taking public transportation (buses, trams, subways)
traffic_topics	train	Bahn <i class=”fa fa-train”></i>	Taking trains
sports_topics	skydiving	Fallschirmspringen	Skydiving
sports_topics	swimming	Schwimmen	Swimming
sports_topics	water_sports	Wassersport (außer Schwimmen, z.B. Segeln, Jetski)	other water sports
sports_topics	motor_sports	Motorsport	Motor sports
sports_topics	shooting_sports	Schießsport	Shooting sports
sports_topics	ski	Skifahren oder ähnlich	Skiing or similar
sports_topics	jogging	Jogging	Jogging
sports_topics	bungee	Bungeejumping	Bungee jumping
sports_topics	mountaineering	Bergsteigen/-wandern	Mountaineering
sports_topics	other_sport	andere	Other sports
safety_topics	frailty	Gebrechlichkeit (z.B. Leiter besteigen)	Frailty (e.g, climbing a ladder)
safety_topics	construction_gardening	Bau-/Gartenarbeiten	Construction and gardening hazards
safety_topics	weapons	Waffen	Weapons
safety_topics	fireworks	Feuerwerk	Fireworks
safety_topics	expose_to_criminals	sich in Gefahr überfallen zu werden begeben	Risking being mugged
safety_topics	going_out_alone	alleine ausgehen	Going out alone
safety_topics	expose_to_terrorism	sich in Terrorgefahr begeben (z.B. öffentliche Plätze)	Risking a terrorist attack (e.g. frequenting public squares)
safety_topics	moral_courage	Zivilcourage zeigen	Showing moral courage
money_topics	bought_home	Haus/-Wohnungskauf, Hausbau	Buying or building a house or apartment
money_topics	sold_home	Haus/-Wohnungsverkauf	Selling a house or apartment
money_topics	found_company	Unternehmen gründen	Found company
money_topics	investment	Investition	Investment
cataclysm_topics	nuclear_accidents	Nukleare Unfälle	Nuclear accidents

Table S4: Available choices in the coding scheme *(continued)*

list_name	name	label	label_en
cataclysm_topics	nuclear_fallout	Radioaktiver Niederschlag	Acid rain
cataclysm_topics	nuclear_waste	Atommüll	Atomic waste
cataclysm_topics	nuclear_war	Atombomben	Nuclear bombs
cataclysm_topics	flooding	Überflutung	Flooding
cataclysm_topics	terror_attack	Terroristischer Angriff	Terrorist attacks
cataclysm_topics	earthquake	Erdbeben	Earthquakes
cataclysm_topics	other_cataclysm	anderes	Other
meaningful_entry	meaningless	Sinnlos	Meaningless
meaningful_entry	nothing	"Keine"/"Nichts"	"None"/"Nothing"
meaningful_entry	nothing_concrete	"An nichts konkretes"	"Nothing concrete"
meaningful_entry	spontaneous	"Spontan"	"Spontaneous"
meaningful_entry	my_behaviour	"Mein Verhalten"	"My behaviour"
meaningful_entry	what_others_tell_me	"was andere mir sagen"	"what others tell me"
meaningful_entry	others_behaviour	"an andere gedacht"	"thought about others"
meaningful_entry	my_feelings	"Meine Gefühle"	"My feelings"
meaningful_entry	other	anderes	other
concreteness	single_concrete	einzelne konkrete Situation an Zeit und Ort	a single concrete situation in time and place
concreteness	multiple_concrete	mehrere konkrete Situationen	several concrete situations
concreteness	behaviour	konkrete Verhaltensweisen, aber unklar wann/wo/wie oft	concrete behaviours, but unclear how often, where, and when
concreteness	specific_topic	spezifisches Themenfeld	specific topic
concreteness	vague_topic	vages Themenfeld	vague topic
worth_it	no_real_answer	keine richtige Antwort	no real answer
worth_it	cant_tell_yet	kann man noch nicht sagen (e.g. in der Zukunft bewertbar)	can't tell yet (e.g., waiting for outcome)
worth_it	not_worth_it	___nicht___ gelohnt	not worth it
worth_it	mixed	gemischt, teils-teils	mixed
worth_it	worth_it	gelohnt	worth it
worth_it	dont_know	weiß nicht	don't know
worth_it	several	mehrere unterschiedliche Antworten	several answers (for different risks)
worth_it	other	andere	other

Table S4: Available choices in the coding scheme *(continued)*

list_name	name	label	label_en
risks_taken	no_avoided	Nein, Risiken, die sie absichtlich nicht eingegangen ist	no, risks that they avoided on purpose
risks_taken	no_others	Nein, Risiken, die andere betrafen	no, risks relating to others
risks_taken	no_unclear	Nein, andere Gründe	no, other reasons
risks_taken	unclear	Unklar	unclear
risks_taken	mixed	Unterschiedlich je nach Unterthema	mixed by subtopic
risks_taken	yes	Ja, selbst eingegangene Risiken	yes, risks they took themselves

S4.3 Rating of risk categories

Table S5: Rating questions to assess risks on 22 characteristics

name	label	choice_low	choice_high
intro	‘r nrow(psytests_assess_risks)+nrow(psytest_risk)‘. Schritt von 5<br>Bitte beurteilen Sie folgendes Risiko.<br>## ”_‘r rated_risk‘_”<br>Wir stellen Ihnen die gleichen Fragen zu sehr unterschiedlichen Risiken. Daher passen die Fragen manchmal nicht perfekt zu dem Risiko. Bitte geben Sie dennoch Ihr Bestes, um die Frage zu beantworten.	NA	NA
	NA	NA	NA
volun	Gehen Menschen die Risiken von ”_‘r rated_risk‘_” freiwillig ein?	freiwillig	unfreiwillig
	Do people face this risk voluntarily?	risk assumed voluntarily	risk assumed involuntarily
immed	In welchem Ausmaß sind die Risiken von ”_‘r rated_risk‘_” unmittelbar - oder sind Konsquenzen erst zu einem späteren Zeitpunkt wahrscheinlich?	sofortiger Effekt	verzögerter Effekt
	To what extent is the risk immediate — or are consequences likely to occur only at some later time?	effect immediate	effect delayed
exposed	Inwieweit sind Risiken von ”_‘r rated_risk‘_” denen bekannt, die ihnen ausgesetzt sind?	Risiken bekannt	Risiken unbekannt
	To what extent are the risks known precisely by the persons who are exposed to those risks?	risk level known precisely	risk level not known

Table S5: Rating questions to assess risks on 22 characteristics *(continued)*

name	label	choice_low	choice_high
science	Inwieweit sind Risiken von ”_‘r rated_risk‘_” der Wissenschaft bekannt?<br>To what extent are the risks known to science?	Risiken bekannt<br>risk level known precisely	Risiken unbekannt<br>risk level not known
control	Wenn Sie den Risiken von ”_‘r rated_risk‘_” ausgesetzt sind, inwieweit können Sie durch Geschick oder Sorgfalt negative Folgen vermeiden?<br>If you are exposed to the risk, to what extent can you, by personal skill or diligence, avoid negative consequences?	Persönliches Risiko kann nicht kontrolliert werden<br>personal risk can’t be controlled	Persönliches Risiko kann kontrolliert werden<br>personal risk can be controlled
newness	Sind die Risiken von ”_‘r rated_risk‘_” neuartig oder alt und vertraut?<br>Is this risk new and novel or old and familiar?	neuartig<br>new	alt<br>old
chronic	Handelt es sich bei den Risiken von ”_‘r rated_risk‘_” um gleichbleibende Folgen (chronisch) oder um katastrophale Folgen?<br>Is this a constant risk with unchanging consequences (chronic) or a catastrophic risk?	chronisch<br>chronic	katastrophisch<br>catastrophic
common	Haben Menschen gelernt mit ”_‘r rated_risk‘_” einigermaßen ruhig und vernünftig umzugehen oder empfinden Menschen große Furcht vor ”_‘r rated_risk‘_” - eine Art schlechtes Bauchgefühl?<br>Is this a risk that people have learned to live with and can think about reasonably calmly, or is it one that people have great dread for—on the level of a gut reaction?	gewöhnlich<br>common	furchterregend<br>dread
conseq	Wenn Menschen aufgrund von ”_‘r rated_risk‘_” etwas Schlimmes passiert, wie wahrscheinlich ist es dann, dass es tödlich endet?<br>When the risk from the activity is realized in the form of a mishap, how likely is it that the consequence will be fatal?	sicher nicht tödlich<br>certain not to be fatal	sicher tödlich<br>certain to be fatal
prevent	Risiko kann entweder durch die Vorbeugung von negativen Konsequenzen oder durch die Verringerung der Schwere der Konsequenzen, nachdem sie auftreten, kontrolliert werden.<br>Inwieweit können Menschen durch persönliche Fähigkeiten oder Fleiß schlimme Konsequenzen von ”_‘r rated_risk‘_” verhindern?<br>Risk can be controlled either by preventing mishaps or by reducing the severity of consequences after they occur. To what extent can people, by personal skill or diligence, prevent mishaps or illnesses from occuring?	sehr verhinderbar<br>much preventive control	kaum verhinderbar<br>little preventive control
severity	Inwieweit kann eine angemessene Maßnahme die Schwere der Konsequenzen von ”_‘r rated_risk‘_” reduzieren?	sehr reduzierbar	kaum reduzierbar

Table S5: Rating questions to assess risks on 22 characteristics *(continued)*

name	label	choice_low	choice_high
	How can proper action reduce the severity of the consequences of X?	severity can’t be controlled	severity can be controlled
exposure	Wie viele Menschen sind den Risiken von ”_‘r rated_risk‘_” in Deutschland ausgesetzt?	wenige	viele
	How many people are exposed to the risks of X in Germany?	few	many
equity	Inwieweit sind Menschen, die ”_‘r rated_risk‘_” ausgesetzt sind, auch die, die profitieren?	die selben	unterschiedliche Menschen
	To what extent are those who are exposed to X the same people as those who receive the benefits?	risks/benefits matched	risks/benefits mismatched
future	Inwiweit birgt ”_‘r rated_risk‘_” Risiken für folgende Generationen?	sehr kleine Bedrohung	sehr große Bedrohung
	To what extent does present pursuit of X pose risks to future generations?	very little threat	very great threat
atwork	Inwieweit sind Menschen den Risiken von ”_‘r rated_risk‘_” bei der Arbeit ausgesetzt?	unwahrscheinlich auf der Arbeit	wahrscheinlich auf der Arbeit
	To what extent are people exposed to the risks of X at work?	unlikely to be exposed at work	likely to be exposed at work
global	Inwieweit kann ”_‘r rated_risk‘_” weltweite Katastrophen und Zerstörung auslösen?	sehr niedriges katastrophales Potential	sehr hohes katastrophales Potential
	To what extent can X cause catastrophes and destruction?	very low catastrophic potential	very high catastrophic potential
observe	Wenn aufgrund von ”_‘r rated_risk‘_” etwas Schlimmes passiert, inwiefern sind die Schäden beobachtbar?	beobachtbar	nicht beobachtbar
	When something bad happens because of X, to what extent is the damage observable?	observable	not observable
changes	Verändern sich die Risiken von ”_‘r rated_risk‘_” über die Zeit?	steigen stark	sinken stark
	Are the risks of X changing?	increasing greatly	decreasing greatly
easered	Wie leicht können Risiken von ”_‘r rated_risk‘_” reduziert werden?	leicht reduzierbar	schwer reduzierbar
	How easily can risks of X be reduced?	easily reduced	not easily reduced
natenv	Sind die Risiken von ”_‘r rated_risk‘_” gefährlicher für Pflanzen und Tiere als für Menschen?	eher eine Bedrohung für Pflanzen und Tiere	eher eine Bedrohung für Menschen

Table S5: Rating questions to assess risks on 22 characteristics *(continued)*

name	label	choice_low	choice_high
	Are the risks of X more of a threat to plants and wildlife than to humans?	more of a threat to plants/wildlife	more of a threat to humans
social	Handelt es sich bei ”_‘r rated_risk‘_” eher um Risiken für Leib und Leben oder für die soziale Position und Beziehungen?	eher für Leib und Leben	eher für die soziale Position und Beziehungen
	Is X rather a risk to life and limb or for social position and relationships?	rather for life and limb	rather for social position and relationships
severalpeople	Birgt ”_‘r rated_risk‘_” nur mögliche Risiken für die Person, die sie eingeht, oder sind auch andere Personen potentiell betroffen?	eine Person	viele andere Personen
	Is X a risk only for the person who takes it, or can others be affected to?	one person	many other persons

Note:
For the 20 items we translated to German, we provide the English originals. For the last two newly formulated questions, we provide our translations from German

S5 Nonresponse analysis

For some responses, it was not possible to code topics. We report reasons topics could not be coded and describe the demographics of nonresponders below. Nonresponse was far higher in SOEP-IS than in BASE-II, probably because BASE-II respondents could take more time to fill out the questionnaires, whereas the computer-assisted personal interviewing used in SOEP-IS could have led to shorter responses.

Non-respondents (and especially respondents who responded briefly without codeable topics) stated lower risk preferences on average, even after adjusting for other demographic differences. This pattern is consistent with people with low risk preferences responding simply that they took "no risks."

Table S6: Reasons topics could not be coded

question	reason	BASE-2	SOEP-IS
Q1	no_text	191 (12%)	460 (24%)
Q1	nothing	37 (2%)	286 (15%)
Q1	nothing_concrete	29 (2%)	75 (4%)
Q1	my_behaviour	4 (0%)	36 (2%)
Q1	meaningless	8 (1%)	17 (1%)
Q1	spontaneous	0 (0%)	16 (1%)
Q1	other	7 (0%)	12 (1%)
Q1	others_behaviour	1 (0%)	3 (0%)
Q1	my_feelings	0 (0%)	0 (0%)
Q1	what_others_tell_me	0 (0%)	0 (0%)
Q2	nothing	222 (14%)	701 (36%)
Q2	no_text	243 (15%)	426 (22%)
Q2	nothing_concrete	14 (1%)	8 (0%)
Q2	meaningless	12 (1%)	6 (0%)
Q2	my_behaviour	3 (0%)	3 (0%)
Q2	spontaneous	0 (0%)	3 (0%)
Q2	other	1 (0%)	1 (0%)
Q2	my_feelings	0 (0%)	0 (0%)
Q2	others_behaviour	0 (0%)	0 (0%)

Note:
Coders noted the reasons certain free-text responses could not be coded for topics. Here, 'no_text' indicates that the question was not answered at all; 'meaningless' indicates the respondent wrote gibberish. The other categories describe brief, often one-word responses that did not mention any specific risks, like writing 'Nothing', 'nothing concrete', 'I thought about my behaviour', 'I responded spontaneously'. Q1 is the question about thoughts while answering the General Risk Question, Q2 is the question about risks taken in the last year.

Table S7: Means and 95% CIs according to response to text questions

Variable	Rated (n=2510)	No response (n=417)	Brief/vague response (n=570)
Years of education	13.64 [13.52;13.76]	12.76 [12.47;13.04]	12.28 [12.04;12.53]
Age	59.09 [58.36;59.82]	60.30 [58.51;62.09]	59.73 [58.20;61.26]
Male	0.48 [0.47;0.50]	0.50 [0.45;0.55]	0.42 [0.37;0.46]
BASE-II	0.54 [0.52;0.56]	0.34 [0.30;0.39]	0.14 [0.12;0.18]
Risk preference	5.19 [5.10;5.28]	4.49 [4.27;4.71]	3.83 [3.64;4.02]
Employment status: employed	0.38 [0.36;0.40]	0.39 [0.34;0.44]	0.35 [0.31;0.39]
Employment status: education/training	0.05 [0.04;0.06]	0.05 [0.03;0.08]	0.04 [0.02;0.05]
Employment status: retired	0.43 [0.41;0.45]	0.42 [0.37;0.47]	0.44 [0.40;0.48]
Employment status: self-employed	0.07 [0.06;0.08]	0.06 [0.04;0.09]	0.04 [0.03;0.06]
Employment status: unemployed	0.07 [0.06;0.08]	0.08 [0.05;0.11]	0.13 [0.11;0.17]
Employment status: unknown/other	0.01 [0.00;0.01]	0.01 [0.00;0.02]	0.00 [0.00;0.01]
Risk preference (adj.)	5.11 [5.02;5.21]	4.47 [4.23;4.70]	3.98 [3.78;4.18]

Note:

For the responder analysis, the people who responded to the free-text questions Q1 and Q2 with codeable topics differed substantially from those who did not respond at all or responded very briefly. Responders were more likely to be BASE-II participants, male, slightly younger, and more educated. There were slight differences in employment status, such as responders being more likely to be employed. Finally, responders stated higher preferences for risk than did nonresponders, even when adjusting for all other demographic covariates (bottom row). Differences were particularly strong when comparing rated responders to those who responded only very briefly and/or vaguely. This pattern is consistent with our suggestion that people who take fewer risks were less likely to mention concrete topics.

S6 Reference frames

For social reference frames, only the SOEP-IS respondents had the option to respond that they answered spontaneously, as well as the option to not respond (as is standard for this panel study). A full third of SOEP-IS respondents said they responded spontaneously and a substantial minority chose not to respond. In the BASE-II study, which offered neither the spontaneous, nor the nonresponse option, average endorsement of all other options was higher and most respondents endorsed two or more options. Nevertheless, the ranking of options was the same across studies. In addition to the differences in the available options, we believe differences between studies could be due to the BASE-II respondents answering questionnaires (and seeing all options simultaneously), whereas SOEP-IS respondents were interviewed using computer-assisted personal interviewing.

On average, BASE-II respondents endorsed more options (mean=2.99) than did SOEP-IS respondents (mean=1.15) in the social/experiential reference frame question. The majority of respondents did not endorse any social reference frame (BASE-II: 48%, SOEP-IS: 12%).

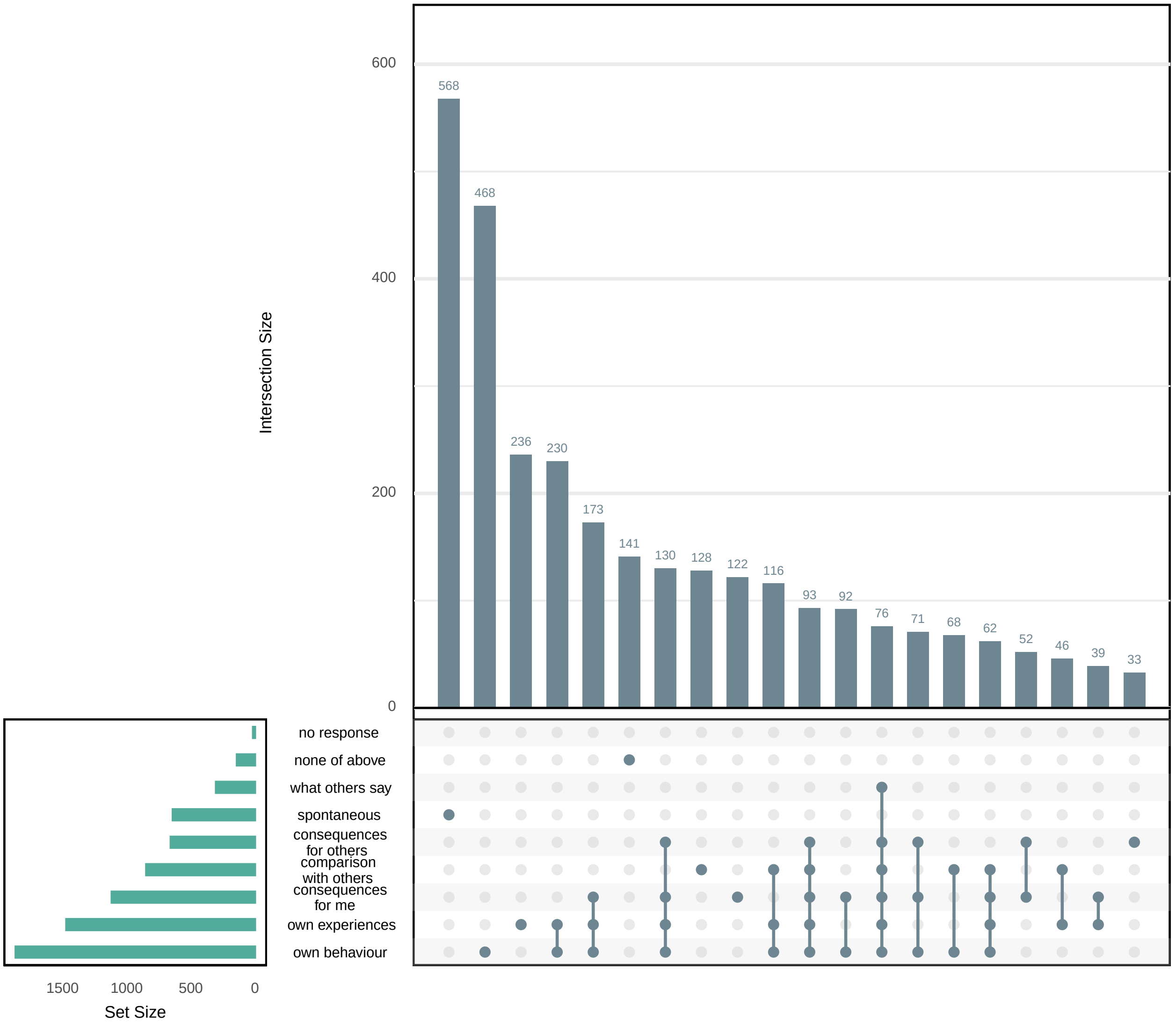

Figure S2: This UpSet plot (Conway et al., 2017) showing the frequency of respondents endorsing one or several options in the question about social, experiential, or behavioural reference frames across the BASE-II and SOEP-IS studies. The lower left panel shows simple counts; the top panel, in combination with the linked dots below it, shows how options were combined.

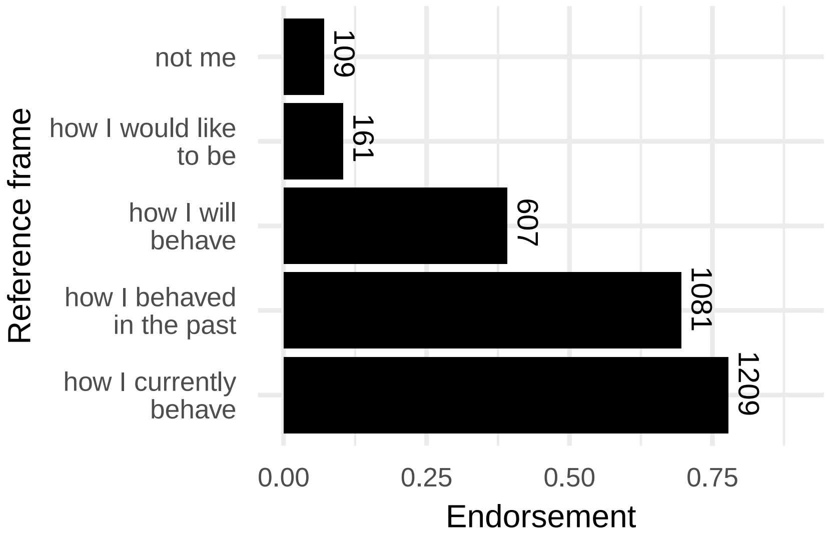

Figure S3: Average endorsement of each temporal reference frame in the BASE-II study. R Respondents could endorse multiple options.

Table S8: Social/experiential reference frame questions

frame	endorsement	n
how I currently behave	78%	1209
how I behaved in the past	70%	1081
how I will behave	39%	607
how I'd like to be	10%	161
not me	7%	109

Note:
On average, BASE-II respondents endorsed more options in the social/experiential reference frame question.

Table S9: Average endorsement of all reference frames by study

frame	BASE-2	SOEP-IS
own_behav	1247 (80%)	617 (32%)
everyday_life	1213 (78%)	n/a
own_exp	1090 (70%)	366 (19%)
past	1088 (70%)	n/a
own_consequences	873 (56%)	251 (13%)
other_comparison	655 (42%)	193 (10%)
future	606 (39%)	n/a
other_consequences	561 (36%)	102 (5%)
other_say	265 (17%)	46 (2%)
ideal	156 (10%)	n/a
not_me	109 (7%)	n/a
no_response	n/a	15 (1%)
none_of_above	n/a	141 (7%)
spontaneous	n/a	636 (33%)

Note:
See S4.1 for more information.

S6.1 By age

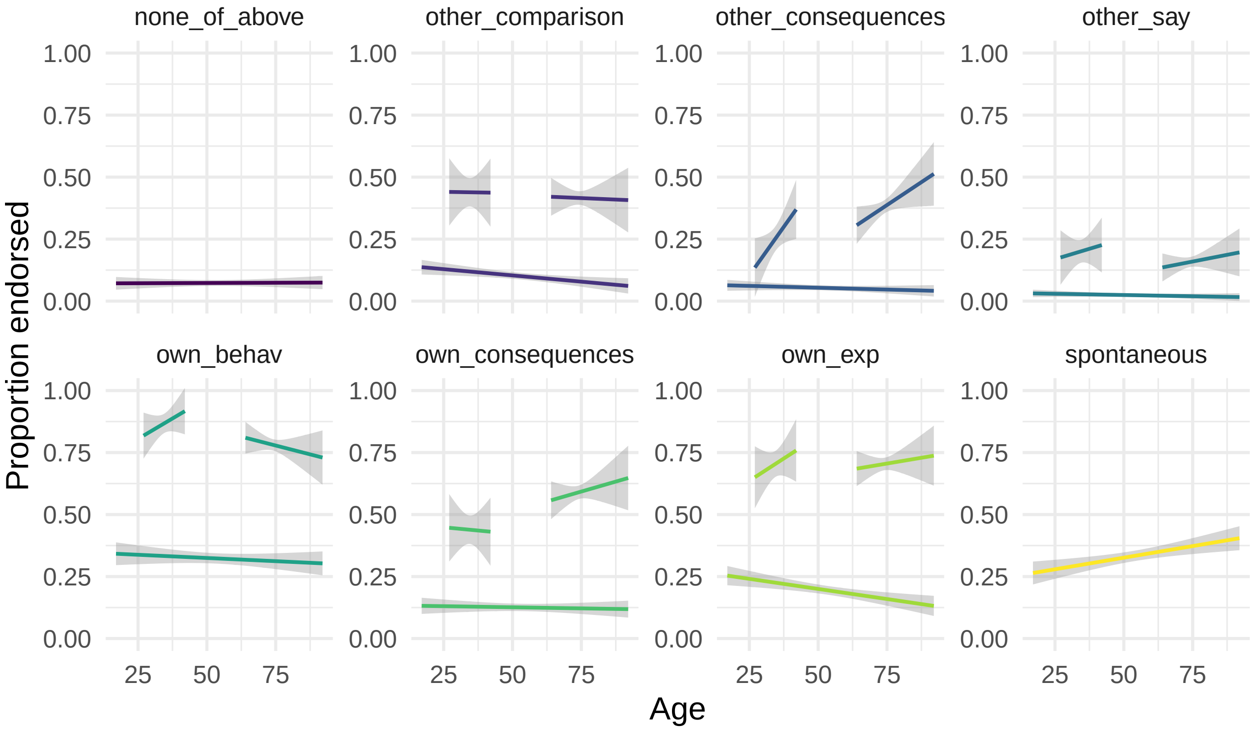

Figure S4: Social/experiential reference frames by age. Interrupted lines show the BASE-II participants, who were split into an older and a younger sample. The continuous lines show the SOEP-IS participants.

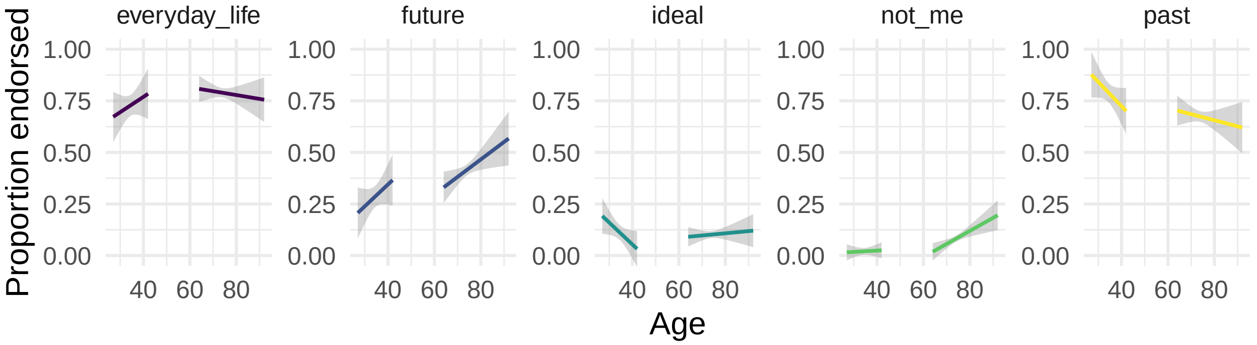

Figure S5: Temporal reference frames by age (BASE-II only). The lines are interrupted because the sampling scheme of BASE-II included an older and a younger subsample.

S6.2 By gender

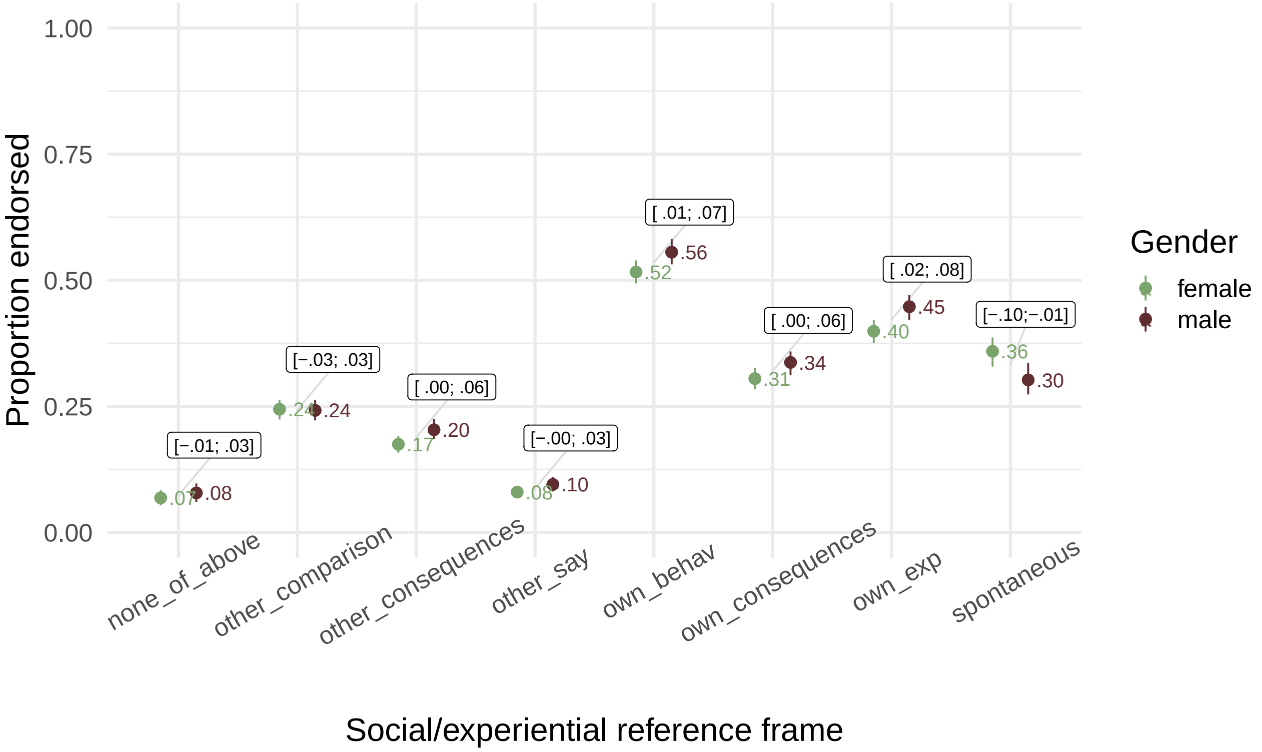

Figure S6: Social/experiential reference frames by gender. Coloured numbers reflect the proportion of each gender that endorsed this reference frame. The numbers in brackets reflect 95% confidence intervals of the difference in proportions.

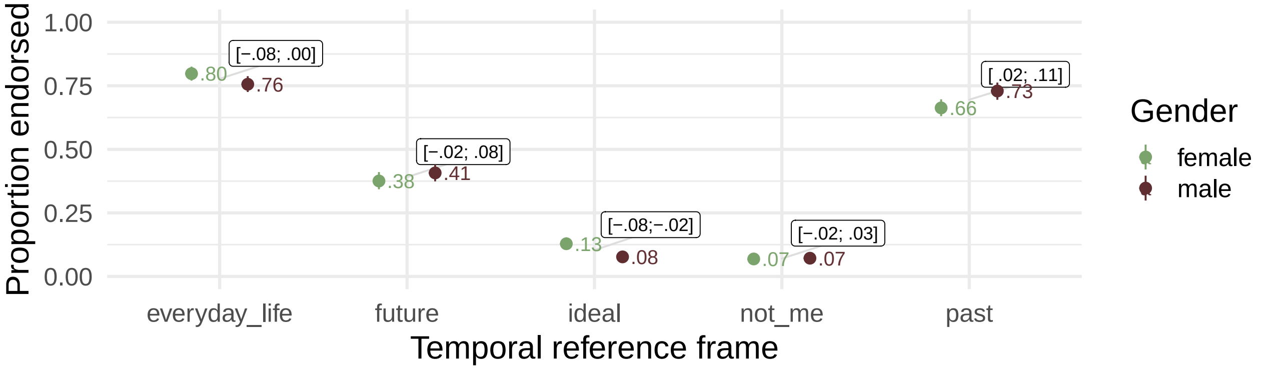

Figure S7: Temporal reference frames by gender (BASE-II only).

S7 Topics

S7.1 Reported topics by question

Table S10: Coded topic mentions in the first free-text question (on what risks people thought about)

topic	n_thoughts	topics
investments	418	investment (115), bought home (53), founded company (12), sold home (6)
relationships	399	moving (76), conflicts (38), children general (33), speaking out (24), separation (19), marriage (16), divorce (12), pregnant (10), colleagues (4), sticking by (4), affairs (3), moving in (3)
traffic	332	car (130), bycycle (76), motorcycle (28), airplane (15), bus (5), train (0)
career	321	
safety	239	frailty (39), construction gardening (27), risking being mugged (23), moral courage (18), going out alone (15), exposure to terrorism (0), fireworks (0), weapons (0)
sports	233	mountaineering (51), skydiving (20), skiing (19), water sports (17), swimming (9), bungee jumping (6), jogging (3), motor sports (1)
travel	212	
other	144	
health	136	operation (24), drinking (11), immediate health risks: other (4), drugs: other (3), unhealthy food (3), other longterm (2), sex (2), smoking (2), cannabis (0), GMO food (0), medication side effects (0)
gambling	60	
crime	15	commit misdemeanors (8), commit crime (2)
cataclysm	10	terror attack (2)

Table S11: Coded topic mentions in the second free-text question (on the biggest risks taken in the last year)

topic	n_last_year	topics
relationships	361	moving (56), conflicts (41), children general (26), speaking out (20), separation (17), pregnant (16), moving in (11), marriage (8), colleagues (6), affairs (4), sticking by (3), divorce (1)
investments	353	investment (127), bought home (33), sold home (7), founded company (3)
traffic	313	car (148), bicycle (96), airplane (18), motorcycle (16), bus (13), train (1)
career	291	
health	235	operation (92), immediate health risks: other (10), other longterm (7), drugs: other (5), sex (5), smoking (5), drinking (4), unhealthy food (4), medication side effects (2), vaccines (1), toxins: other (0), vaccine avoidance (0)
travel	221	
safety	198	construction gardening (48), frailty (46), going out alone (21), moral courage (13), risking being mugged (11), exposure to terrorism (3), fireworks (0), weapons (0)
sports	181	mountaineering (49), water sports (19), skiing (14), swimming (10), jogging (4), skydiving (3), bungee jumping (2), motor sports (0), shooting sports (0)
other	85	
gambling	61	
crime	22	commit misdemeanors (10), commit crime (2)
cataclysm	4	earthquake (1), terror attack (1), flooding (0), nuclear waste (0)

S7.2 Combinations

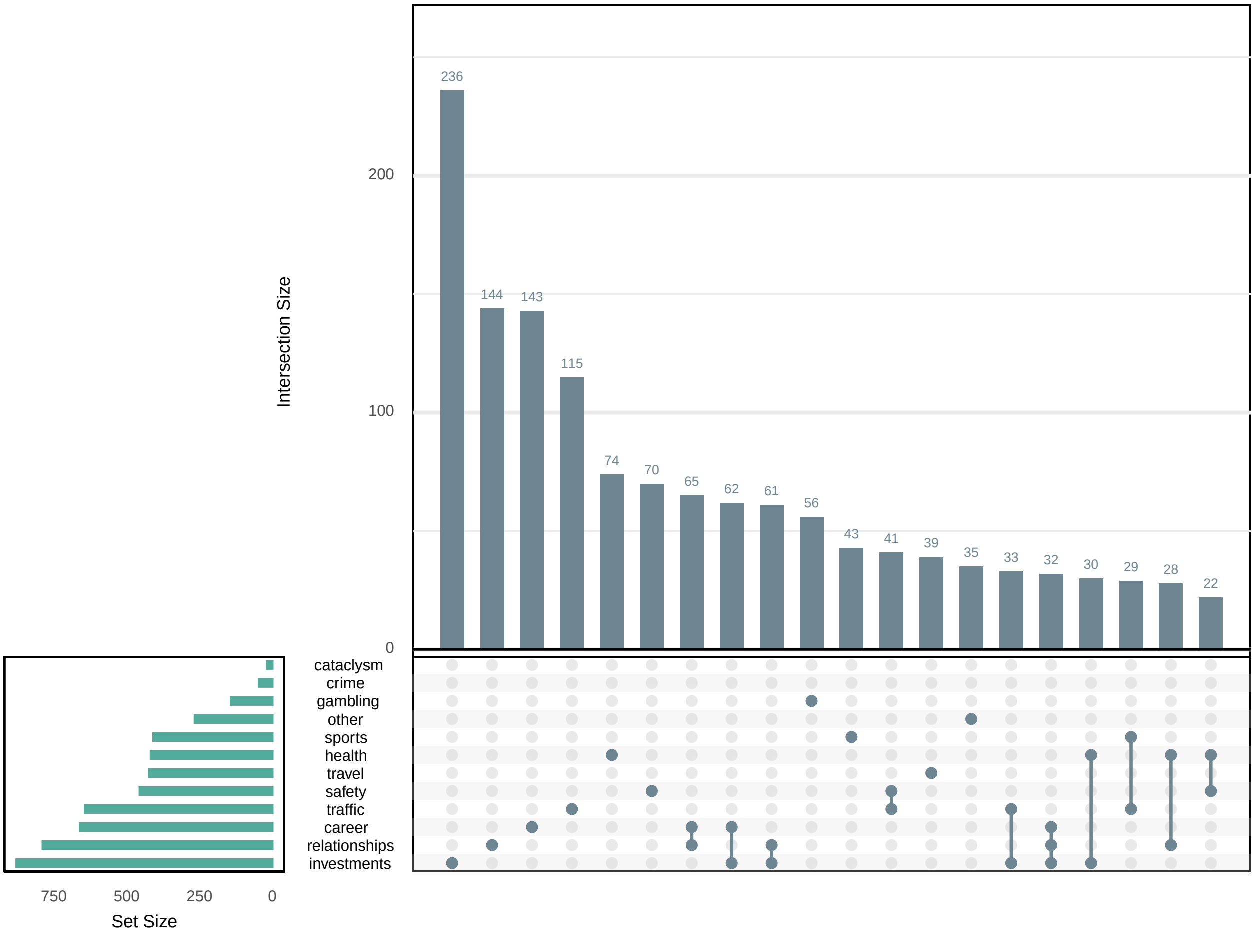

Figure S8: UpSet plot (Conway et al., 2017) showing the frequency with which topics were mentioned (lower left green plot) and how often certain combinations of topics were mentioned (top right blue plot).

S7.3 Detail level

Table S12: Specificity of topics

question	reason	BASE-2	SOEP-IS
Q1	specific topic	243 (26%)	250 (37%)
Q1	unknown time and place but concrete behaviour	416 (45%)	220 (32%)
Q1	vague topic	70 (8%)	120 (18%)
Q1	single concrete situation	87 (9%)	80 (12%)
Q1	multiple concrete situations	113 (12%)	11 (2%)
Q2	unknown time and place but concrete behaviour	460 (56%)	260 (42%)
Q2	specific topic	107 (13%)	156 (25%)
Q2	single concrete situation	234 (29%)	149 (24%)
Q2	vague topic	20 (2%)	47 (8%)

Note:
Coders noted whether the topics mentioned were vague (e.g., health), specific (e.g., buying property), concrete behaviours but with no specified time or place (e.g., ’riding horses without a helmet’), or concrete behaviours with a specified time and/or place (e.g., last winter I tried a very dangerous ski run). Percentages as a fraction of all who gave a codeable response.

S7.4 Age trends

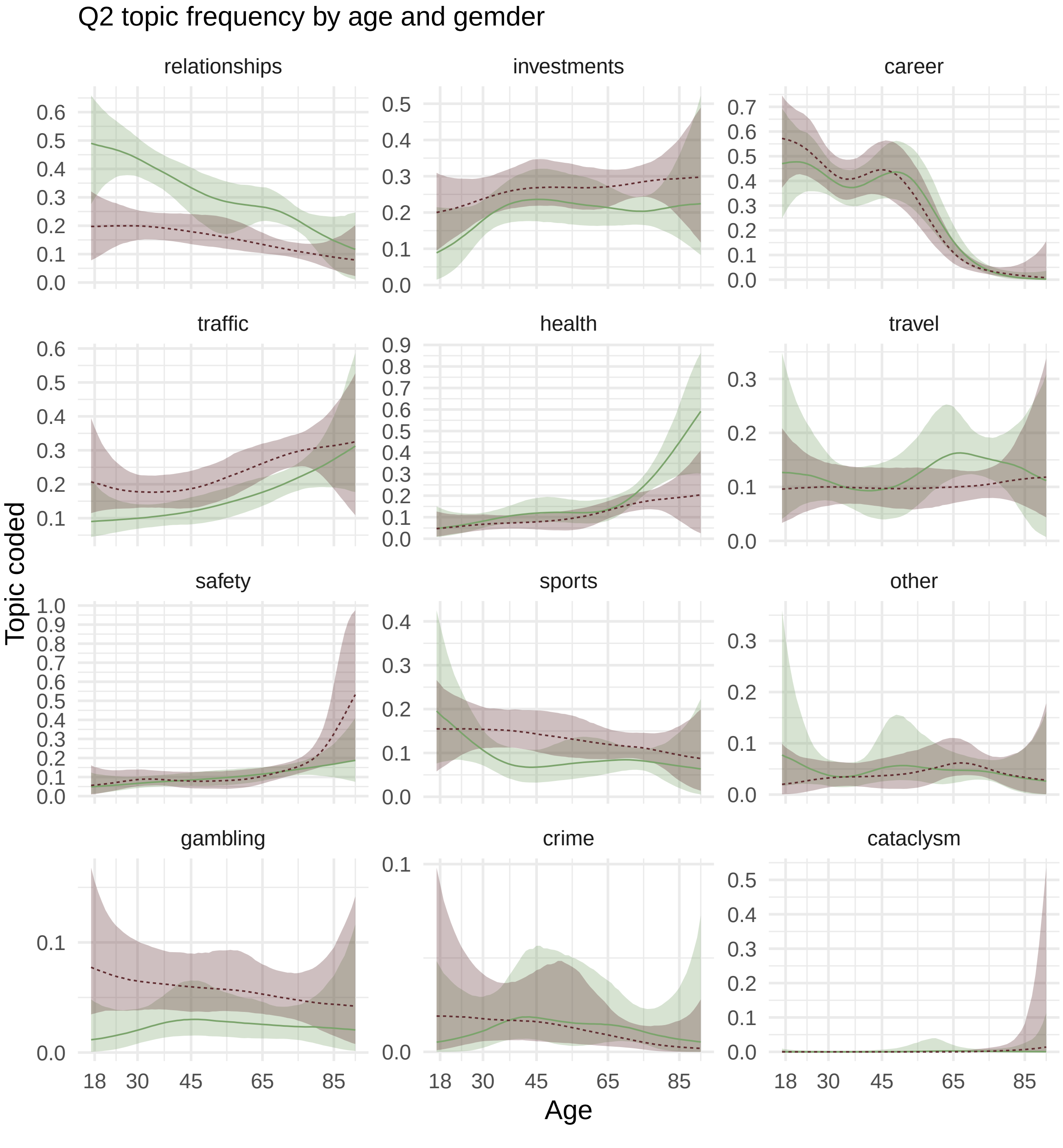

Figure S9: Age trends in mentioning risk domains in the response to the second question (about the biggest risks taken in the past year). The lines show local polynomial regression fits estimated separately by gender in logistic regressions (with shaded 95% confidence intervals). Solid green lines refer to women, dashed red lines refer to men.

Table S13: Question 2 model weights for age and gender effects

topic	no_chg	age_diff	gender_diff	gender_age	gender_x_age
career	0	100	0	0	0
cataclysm	0	100	0	0	0
crime	84	16	0	0	0
gambling	11	0	89	0	0
health	12	0	0	28	60
investments	0	17	35	48	0
other	96	4	0	0	0
relationships	4	0	0	91	5
safety	11	89	0	0	0
sports	0	12	58	1	29
traffic	0	8	10	82	0
travel	21	0	45	2	32

Note:
We compared four models for each topic using approximative leave-one-out cross-validation (LOO-IC) and derived model weights, which index how strongly each model should contribute to predictions of held-out data. We did not find strong evidence for gender differences in age trends for any topic.

Table S14: Question 1 model weights for age and gender effects

topic	no_chg	age_diff	gender_diff	gender_age	gender_x_age
career	0	16	2	65	17
cataclysm	23	34	0	0	43
crime	63	0	0	0	37
gambling	12	9	0	15	64
health	18	82	0	0	0
investments	0	59	0	0	41
other	29	67	0	0	4
relationships	0	3	55	15	27
safety	22	20	0	0	58
sports	6	34	0	8	51
traffic	2	7	77	14	0
travel	0	10	62	27	0

Note:
We compared four models for each topic using approximative leave-one-out cross-validation (LOO-IC) and derived model weights, which index how strongly each model should contribute to predictions of held-out data. We did not find strong evidence for gender differences in age trends for any topic.

S7.5 Multiple imputation in case of nonresponse

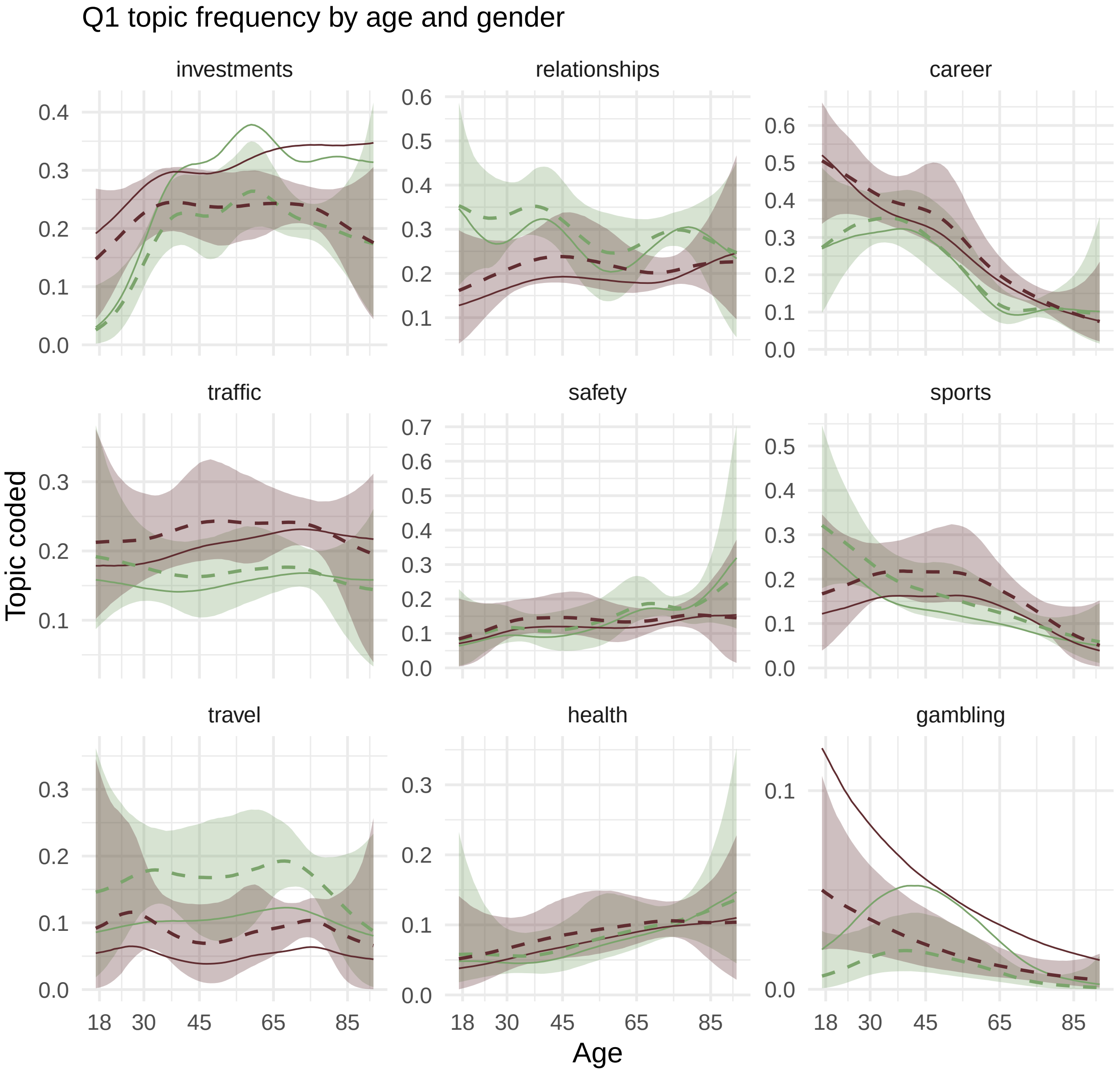

Figure S10: Age trends by gender in mentioning risk domains in the response to the first question (about what people thought about). We altered Figure 5 and added dashed lines to show fit lines with 95% CIs estimated based on 10-fold multiple imputed data. We included age, gender, years of education, stated risk preference, coder ratings, coder confidence, number of topics in Q1 and Q2, the text length, and coded topics in Q1 and Q2 in the imputation model. We also included third-order polynomial terms for age and their interaction with gender. The topics crime, cataclysm, and other were excluded before multiple imputation to reduce multicollinearity and because they were rare. We verified the convergence of the imputation via visual diagnostics. Multiple imputation mainly led to slightly changed averages for several topics, but not to qualitatively different age and gender differences.

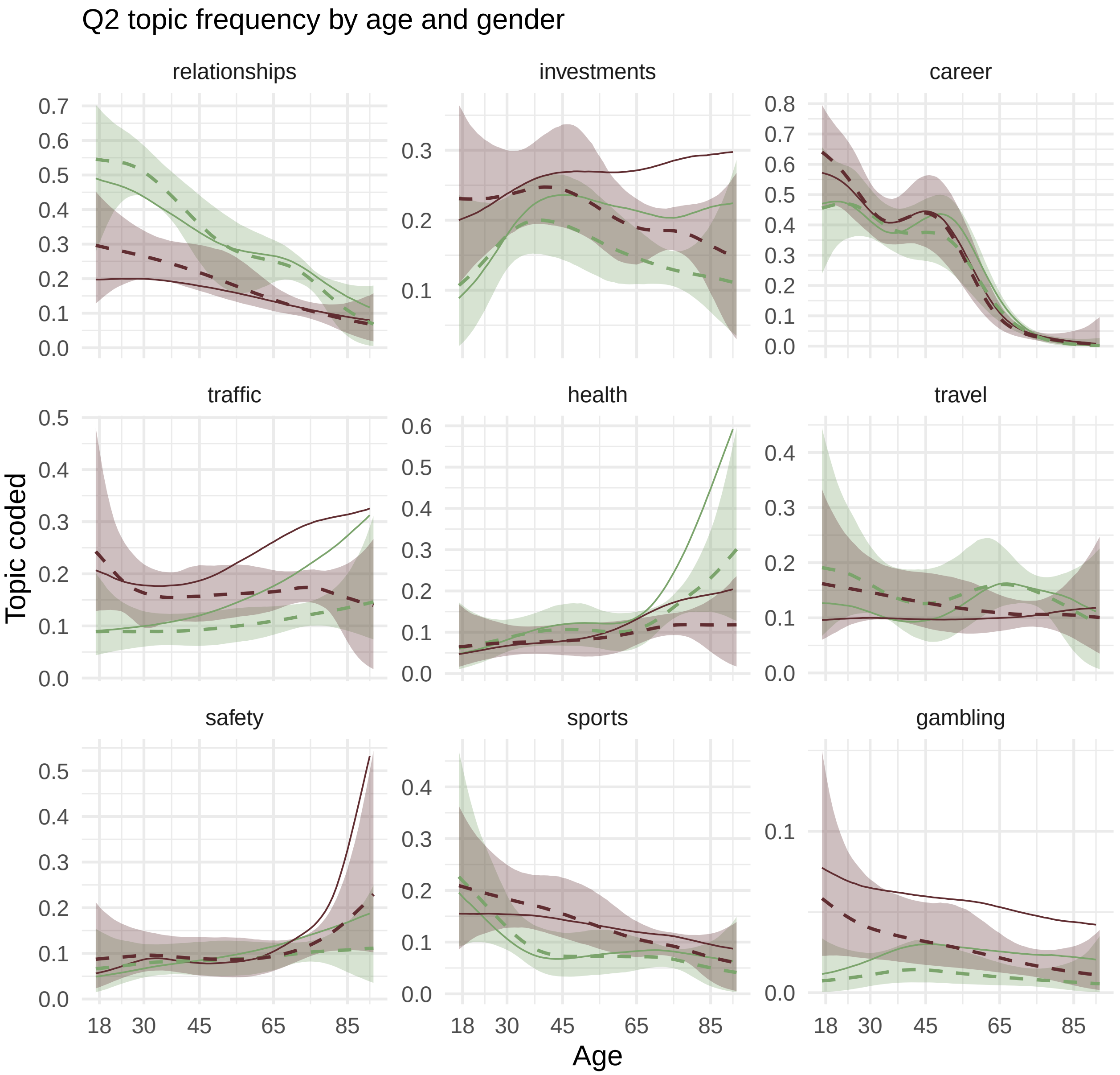

Figure S11: This graph again shows age trends by gender in mentioning risk domains in the response to the second question (about the biggest risks taken in the past year). We altered Figure S9 and added dashed lines to show fit lines with 95% CIs estimated based on 10-fold multiple imputed data. We included age, gender, years of education, stated risk preference, coder ratings, coder confidence, number of topics in Q1 and Q2, the text length, and coded topics in Q1 and Q2 in the imputation model. We also included third-order polynomial terms for age and their interaction with gender. The topics crime, cataclysm, and other were excluded before multiple imputation to reduce multicollinearity and because they were rare. We verified the convergence of the imputation via visual diagnostics. Multiple imputation mainly led to slightly changed averages for several topics, but not to qualitatively different age and gender differences.

S7.6 Gender differences

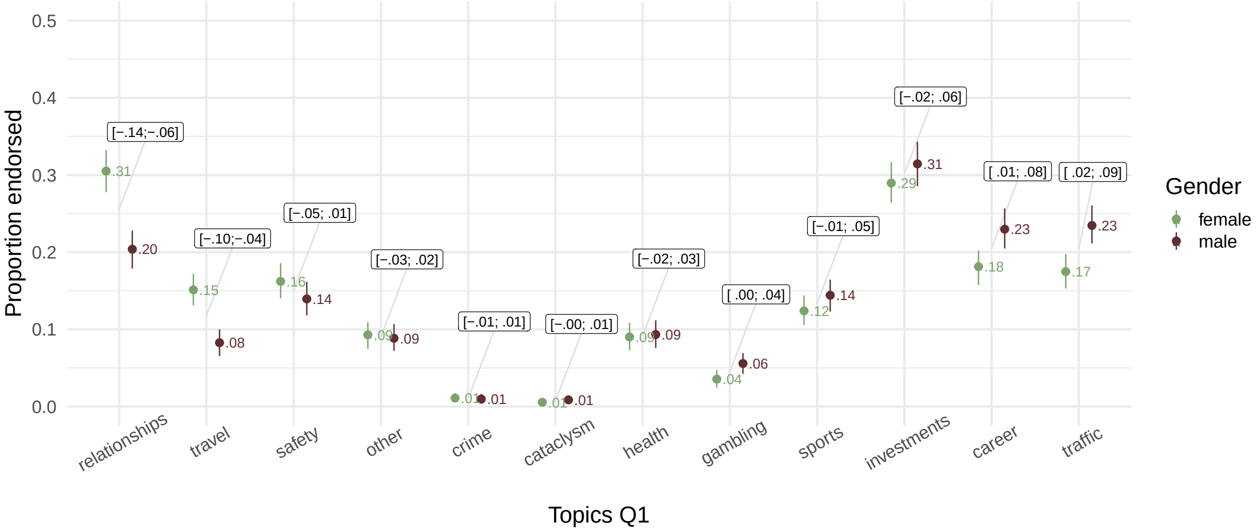

Figure S12: Topics in response to Q1 by gender, pooled across age. Coloured numbers reflect the proportion by each gender mentioning this topic. Numbers in brackets reflect 95% confidence intervals of the difference in proportions.

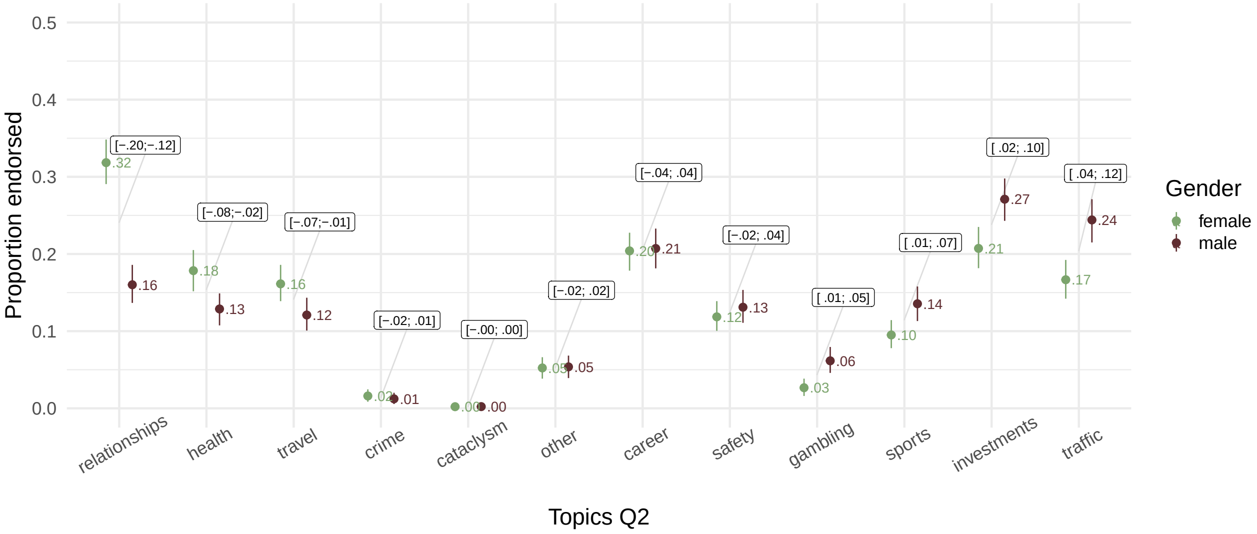

Figure S13: Topics in response to Q2 by gender, pooled across age. Coloured numbers reflect the proportion by each gender mentioning this topic. Numbers in brackets reflect 95% confidence intervals of the difference in proportions.

S7.7 Word clouds

We used the pipeline documented in https://osf.io/aj3bn/wiki/home/ to preprocess the texts written in response to the questions (i.e., tokenisation, spelling correction, stop word removal, stemming, translation) and generate unigram and bigram word clouds.

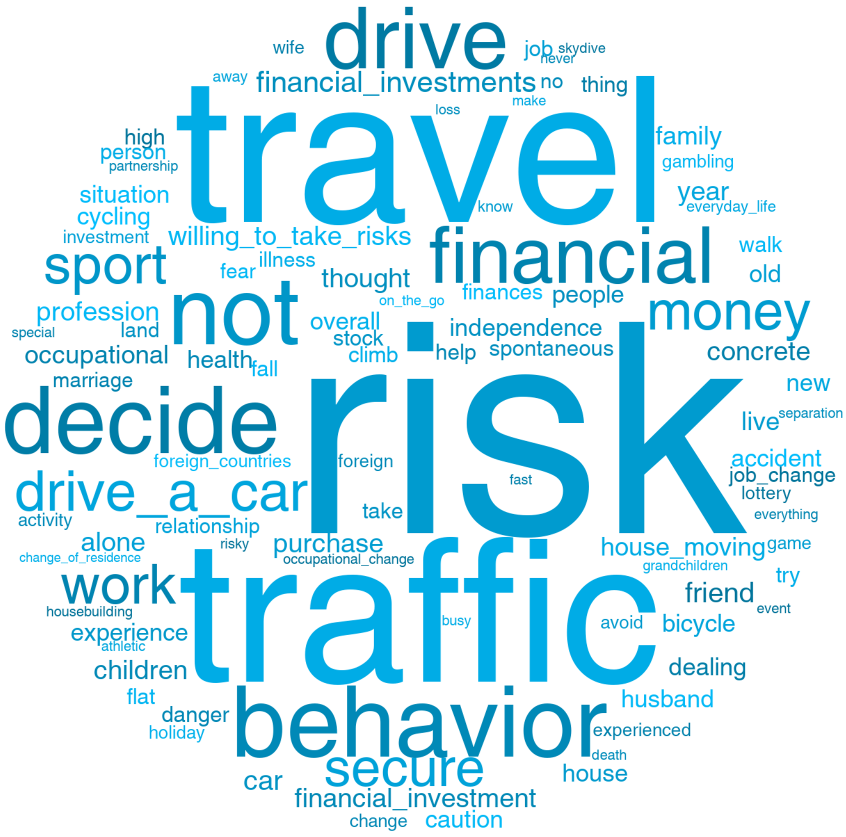

Figure S14: Inverse-document-frequency-weighted word cloud showing common single words in sizes proportional to their frequency in the responses to the first free-text question (on what people thought about). Unigrams were counted in German and then translated; some displayed terms therefore contain more than one word.

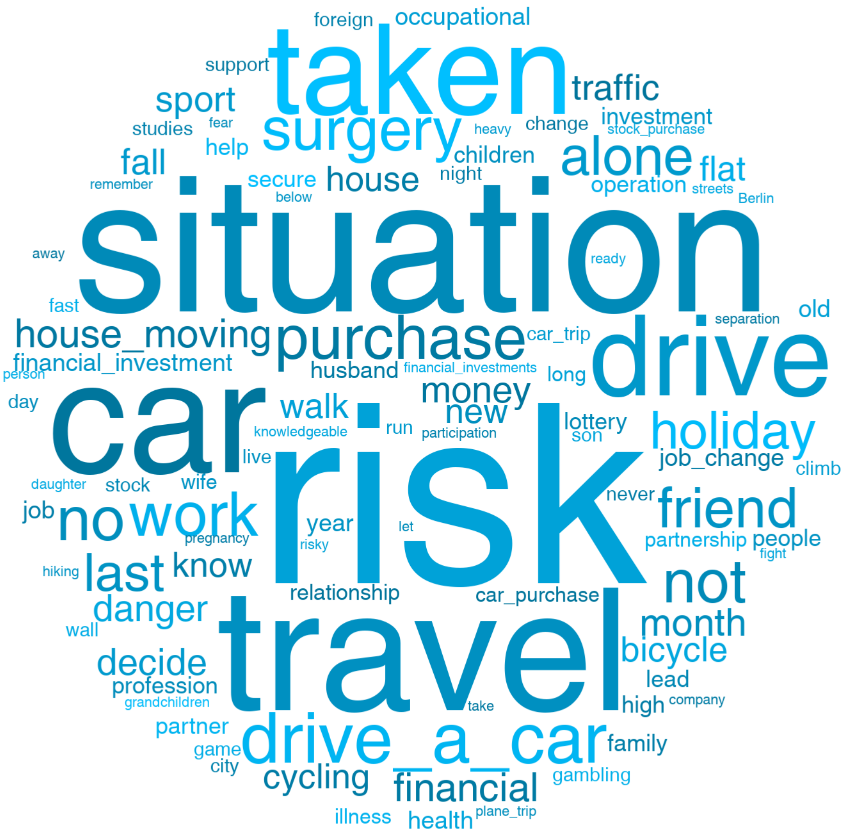

Figure S15: Inverse-document-frequency-weighted word cloud showing single words in sizes proportional to their frequency in the responses to the second free-text question (on the biggest risks taken in the last year). Unigrams were counted in German and then translated; some displayed terms therefore contain more than one word.

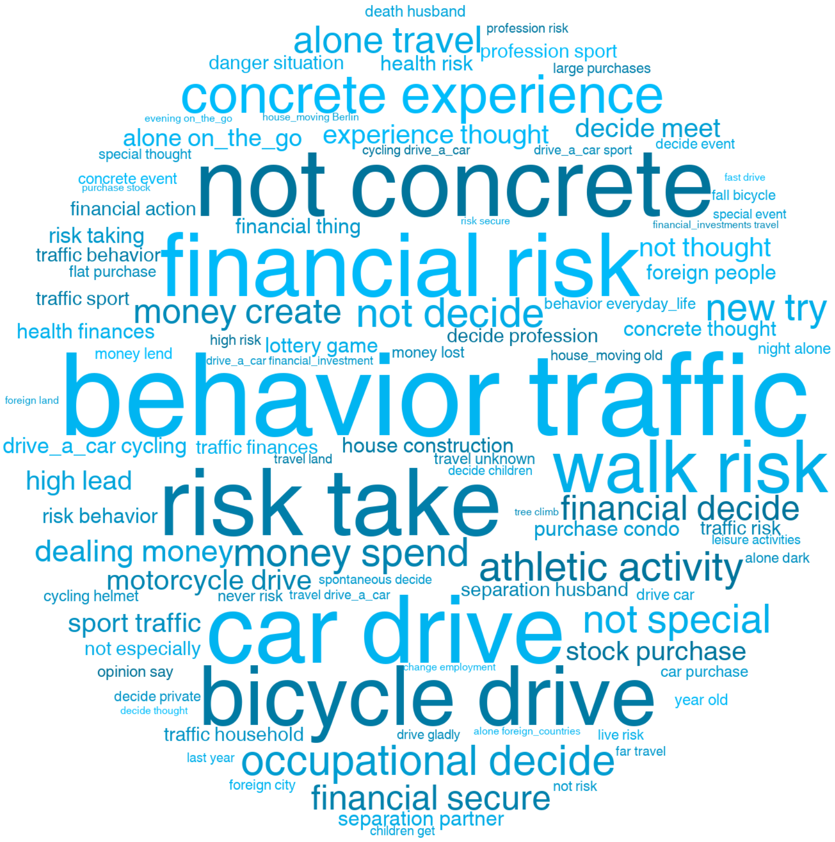

Figure S16: Inverse-document-frequency-weighted word cloud showing common bigrams in sizes proportional to their frequency in the responses to the first free-text question (on what people thought about). Bigrams were counted in German and then translated; some displayed terms therefore contain more than two words.

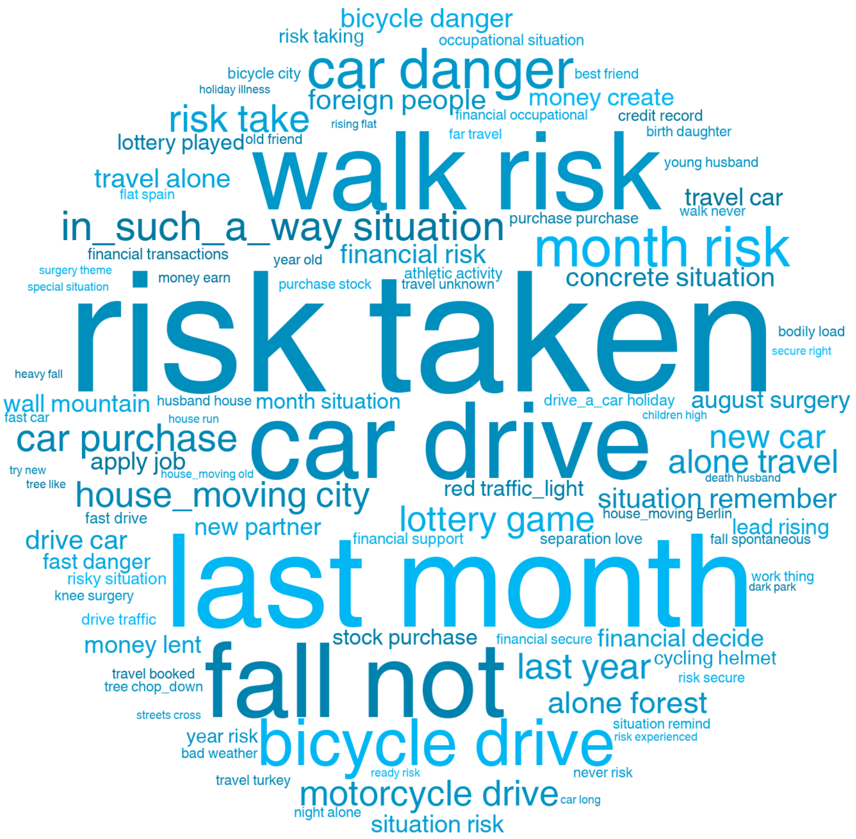

Figure S17: Inverse-document-frequency-weighted word cloud showing common bigrams in sizes proportional to their frequency in the responses to the second free-text question (on the biggest risks taken in the last year). Bigrams were counted in German and then translated; some displayed terms therefore contain more than two words.

S8 Quantifying risks according to psychometric characteristics

S8.1 Agreement across raters

Table S15: Intra-class correlations (ICCs) for risk characteristics

variable	label	ICC
social	Is X rather a risk to life and limb or for social position and relationships?	0.97
conseq	When the risk from the activity is realized in the form of a mishap, how likely is it that the consequence will be fatal?	0.96
global	To what extent can X cause catastrophes and destruction?	0.95
volun	Do people face this risk voluntarily?	0.94
common	Is this a risk that people have learned to live with and can think about reasonably calmly, or is it one that people have great dread for—on the level of a gut reaction?	0.93
future	To what extent does present pursuit of X pose risks to future generations?	0.93
immed	To what extent is the risk immediate — or are consequences likely to occur only at some later time?	0.93
severalpeople	Is X a risk only for the person who takes it, or can others be affected to?	0.92
atwork	To what extent are people exposed to the risks of X at work?	0.91
exposure	How many people are exposed to the risks of X in Germany?	0.90
prevent	Risk can be controlled either by preventing mishaps or by reducing the severity of consequences after they occur. To what extent can people, by personal skill or diligence, prevent mishaps or illnesses from occuring?	0.89
control	If you are exposed to the risk, to what extent can you, by personal skill or diligence, avoid negative consequences?	0.86
natenv	Are the risks of X more of a threat to plants and wildlife than to humans?	0.86
chronic	Is this a constant risk with unchanging consequences (chronic) or a catastrophic risk?	0.85
equity	To what extent are those who are exposed to X the same people as those who receive the benefits?	0.84
easered	How easily can risks of X be reduced?	0.82
exposed	To what extent are the risks known precisely by the persons who are exposed to those risks?	0.81
severity	How can proper action reduce the severity of the consequences of X?	0.81
newness	Is this risk new and novel or old and familiar?	0.80
observe	When something bad happens because of X, to what extent is the damage observable?	0.79
changes	Are the risks of X changing?	0.73
science	To what extent are the risks known to science?	0.73

Note:
These ICCs quantify interrater agreement on the placement of risk factors on characteristic dimensions. Each online rater rated 3-5 risk topics on all characteristics. All risk topics were rated by at least 17 raters except two (where we split two related topics). To obtain the reliability of the averaged ratings, we used the Spearman-Brown prophecy formula with the minimum of 17 raters.

S8.2 Confirmatory factor analysis

We ran a confirmatory factor analysis on the average ratings of 63 risks on 16 characteristics to extract the factors *dread* and *unknown*, which we defined following Slovic (1987). The factor *dread* was allowed to load on the items *global*, *severity*, *changes*, *control* (R), *common* (R), *conseq*, *easered* (R), *equity* (R), *future*, *volun* (R), and *chronic* (R). The factor *unknown* was allowed to load on the items *newness*, *science* (R), *observe* (R), *exposed* (R), and *immed* (R). (R) indicates items loading in reverse. The texts for the items can be found in Table S5 above and in Figure S18 (English translations only). The reliability (coefficient omega) of the factors was *dread*: 0.92 and *unknown*: 0.81. The two factors were moderately correlated (r=0.43 [0.20;0.61]).

The following output shows the model fit indicators and factor loadings as calculated by the R package lavaan.

```
## lavaan 0.6-4 ended normally after 32 iterations
##
##   Optimization method                           NLMINB
##   Number of free parameters                         33
##
##   Number of observations                            63
##
##   Estimator                                         ML
##   Model Fit Test Statistic                     558.758
##   Degrees of freedom                               103
##   P-value (Chi-square)                           0.000
##
## Parameter Estimates:
##
##   Information                                 Expected
##   Information saturated (h1) model          Structured
##   Standard Errors                             Standard
##
## Latent Variables:
##                    Estimate  Std.Err  z-value  P(>|z|)   Std.lv  Std.all
##   dread =~
##     global             1.31     0.15     9.02     0.00     1.31     0.89
##     severity           0.58     0.08     6.99     0.00     0.58     0.76
##     changes            0.28     0.06     4.90     0.00     0.28     0.58
##     controlR           0.79     0.10     7.87     0.00     0.79     0.82
##     commonR            0.96     0.13     7.34     0.00     0.96     0.78
##     conseq             0.64     0.16     4.09     0.00     0.64     0.49
##     easeredR           0.66     0.08     8.05     0.00     0.66     0.83
##     equityR            0.83     0.11     7.66     0.00     0.83     0.81
##     future             1.06     0.14     7.31     0.00     1.06     0.78
##     volunR             1.06     0.14     7.34     0.00     1.06     0.78
##     chronicR           0.41     0.11     3.64     0.00     0.41     0.45
##   unknown =~
##     newness            0.51     0.09     5.85     0.00     0.51     0.67
##     scienceR           0.37     0.07     5.50     0.00     0.37     0.64
##     observeR           0.49     0.08     5.90     0.00     0.49     0.68
##     exposedR           0.74     0.08     9.79     0.00     0.74     0.97
##     immedR             0.79     0.15     5.17     0.00     0.79     0.61
##
## Covariances:
##                    Estimate  Std.Err  z-value  P(>|z|)   Std.lv  Std.all
##   dread ~~
```

```
##     unknown             0.41     0.11     3.58     0.00     0.41     0.41
##
## Variances:
##                     Estimate  Std.Err  z-value  P(>|z|)   Std.lv  Std.all
##    .global              0.43     0.10     4.33     0.00     0.43     0.20
##    .severity            0.25     0.05     5.18     0.00     0.25     0.43
##    .changes             0.16     0.03     5.45     0.00     0.16     0.67
##    .controlR            0.30     0.06     4.94     0.00     0.30     0.33
##    .commonR             0.57     0.11     5.10     0.00     0.57     0.39
##    .conseq              1.28     0.23     5.51     0.00     1.28     0.76
##    .easeredR            0.19     0.04     4.88     0.00     0.19     0.31
##    .equityR             0.37     0.07     5.01     0.00     0.37     0.35
##    .future              0.71     0.14     5.10     0.00     0.71     0.39
##    .volunR              0.71     0.14     5.10     0.00     0.71     0.39
##    .chronicR            0.67     0.12     5.53     0.00     0.67     0.80
##    .newness             0.32     0.06     5.19     0.00     0.32     0.55
##    .scienceR            0.20     0.04     5.28     0.00     0.20     0.59
##    .observeR            0.28     0.05     5.18     0.00     0.28     0.54
##    .exposedR            0.04     0.04     0.90     0.37     0.04     0.06
##    .immedR              1.07     0.20     5.35     0.00     1.07     0.63
##     dread               1.00                                1.00     1.00
##     unknown             1.00                                1.00     1.00
```

S8.3 Rated item means

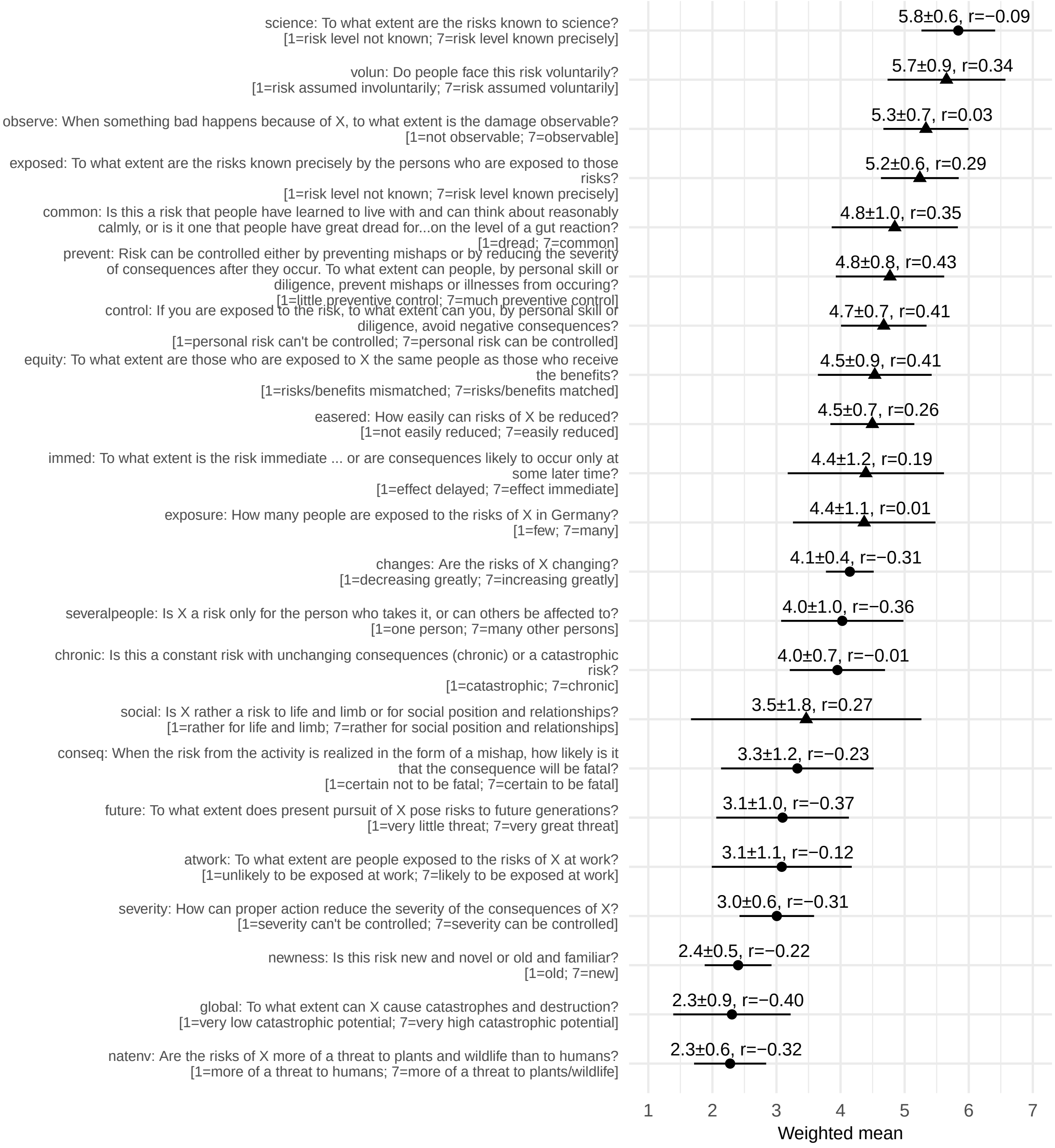

Figure S18: Rated item means and rank correlations. The factors *dread* and *unknown* were used to give a high-level summary of how the risks scored on these 22 characteristics. Here, we wanted to show how highly the risks people mentioned ranked on each characteristic on average and how the average on the characteristic related to how frequently risks were mentioned. We therefore log+1-transformed the frequencies of each risk and calculated frequency-weighted means and standard deviations of all risks on all characteristics, as well as Spearman rank correlations with frequency.

S9 Can coders predict risk preference from the text?

S9.1 Unmasking

Coders had noted when gender, age, residence or other identifying characteristics were apparent from the text. We wanted coders to base their inference about respondents' risk preferences on the text's content, not on stereotypes about men and women, or old and young. Therefore, we had coders note when respondents identified themselves through their responses. In total, there were 62 (3%) individuals with information that could indicate their gender, age, or residence.

We found little evidence that coders used unmasking information gleaned from the text (e.g, when gender or age were apparent from the text) for their ratings (i.e., adjusting for unmasking did not attenuate the accuracy coefficient, nor did excluding unmasked texts attenuate the coefficient), and they did not do so in the expected, stereotypical way (i.e., raters estimated a higher average risk preference for respondents who identified themselves as women, even though this runs counter to population differences). Still, we omitted any texts where personal information was apparent according to at least two coders.

Because texts might also contain indirect hints about gender and age, we also conducted an analysis of rater accuracy while adjusting for real (not inferred) gender and age. Again, the coefficient indexing accuracy was not attenuated and coders did not give men higher ratings on average.

Table S16: Unmasking effects on coder ratings

term	estimate	conf.low	conf.high
(Intercept)	-0.01	-0.05	0.03
unmasking_female1	0.45	0.11	0.79
unmasking_male1	-0.01	-0.55	0.54
unmasking_age1	0.59	-0.28	1.47

Note:
Coder ratings ran counter to stereotypes (unmasked women and older people were given slightly higher ratings). Standardised regression coefficients with 95% confidence intervals (CI).

Table S17: Attenuation of accuracy

term	estimate	conf.low	conf.high
(Intercept)	-0.06	-0.19	0.08
risk_gen	0.28	0.24	0.32
male	-0.01	-0.09	0.07
age	0.00	0.00	0.00

Note:
There was no attenuation of coder accuracy when adjusting for real gender and age of respondent. Standardised regression coefficients with 95% confidence intervals (CI).

Table S18: Including unmasked individuals

term	estimate	conf.low	conf.high
(Intercept)	-0.04	-0.08	0.00
risk_gen	0.28	0.24	0.32

Note:
There was very little difference in accuracy when texts with unmasking information were included (rather than excluded, as was the case for all following analyses). Standardised regression coefficients with 95% confidence intervals (CI).

S9.2 Rank-order and mean differences

The averaged coder rating predicted the self-rated general risk preference with a correlation (95% confidence interval) of 0.27 [0.23; 0.31] (Spearman rank correlation: r=0.27). The coders estimated a mean of 5.04, whereas self-ratings averaged at 5.18. Standard deviations (SD) differed more. Coder ratings had an SD of 1.51, whereas self-ratings had an SD of 2.3. The coder ratings are the average of three ratings. This reduces the SD from 1.78 (square root of the averaged variances across coders). SDs for individual coders ranged from 1.2 to 2.6. When restricting the sample to cases where coders indicated a higher confidence than 1 (on a scale of 0 to 3), the mean difference was reduced to 0.08 [-0.02; 0.19].

S9.2.1 Previous waves

Participants in both SOEP-IS and BASE-II had answered the GRQ in previous years of the longitudinal studies. We averaged 2.12 different self-reports/years from n=1938 individuals. The average from previous waves (GRQp) correlated substantially with the self-report in the most recent wave (GRQ 0.56 [0.53; 0.59]).

The correlation between GRQp and coder-rated risk preference (r=0.15 [0.11; 0.19]) was lower than the correlation between GRQ and coder-rated risk preference (0.27 [0.23; 0.31]).

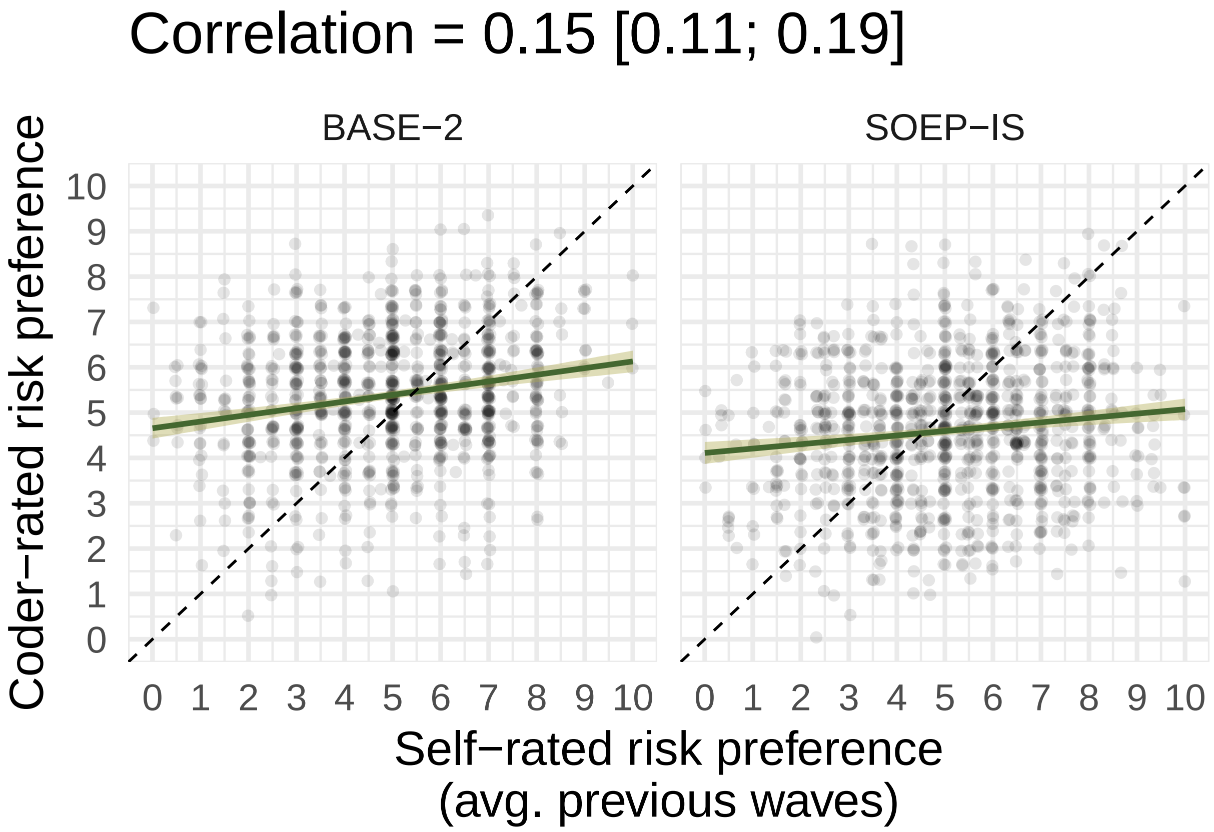

Figure S19: Coder ratings and stated preferences from previous waves.

S9.3 Linearity

We wanted to test whether the relationship between stated preferences and coder ratings is approximately linear. Visual inspection and an approximative leave-one-out-adjusted (LOO-IC) model comparison are both consistent with a linear fit.

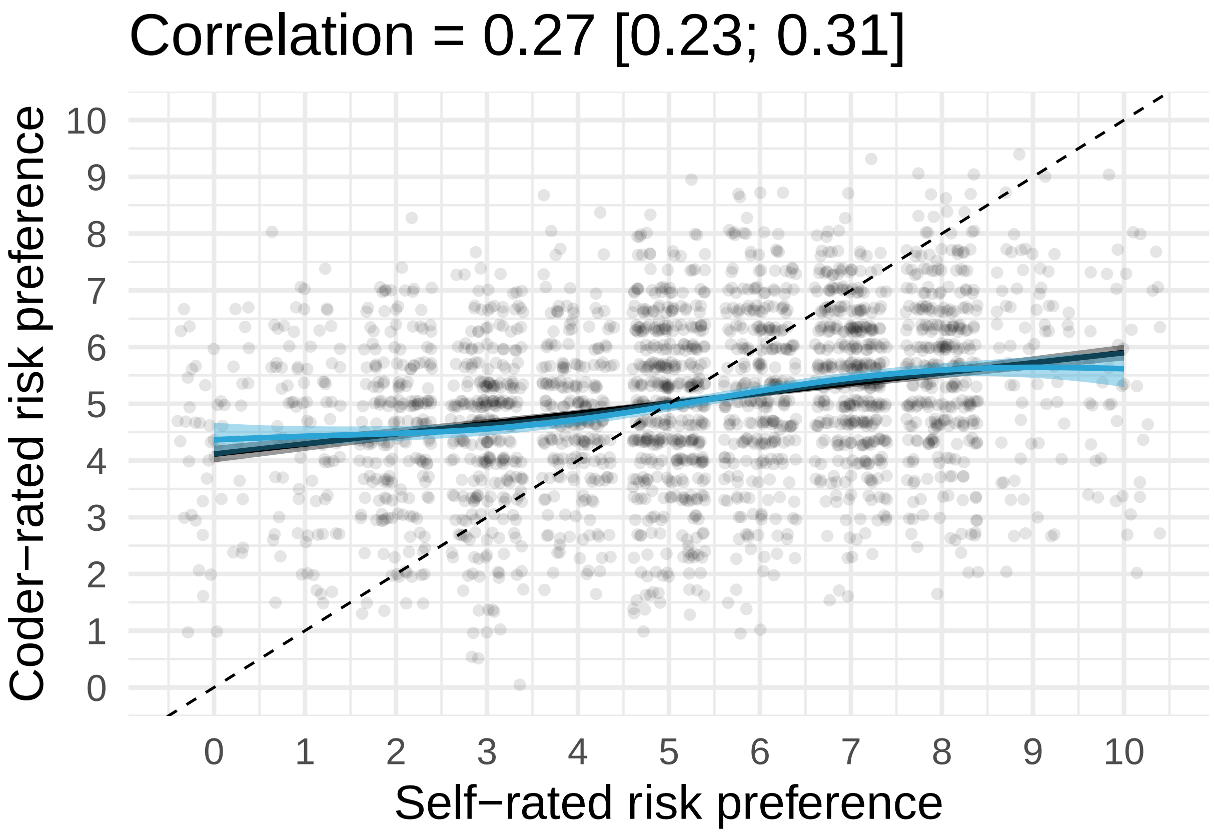

Figure S20: Testing whether the relationship between stated preferences and coder rating is linear. The blue line shows the best fit of a generalized additive model with a thin-plate spline; the black line shows a linear fit.

Table S19: Linearity model comparison

	LOOIC	SE
m__accuracy - m__accuracy__nonlinear	6.85	6.4
m__accuracy - m__accuracy__discretised	0.92	9.6
m__accuracy__nonlinear - m__accuracy__discretised	-5.92	4.5

Note:
Comparison of a simple linear model to a model with a thin-plate spline and a model with a discretised risk preference variable. The simple model fits almost as well (within 2 standard errors of the approximative leave-one-out information criterion).

S9.4 Differences by study

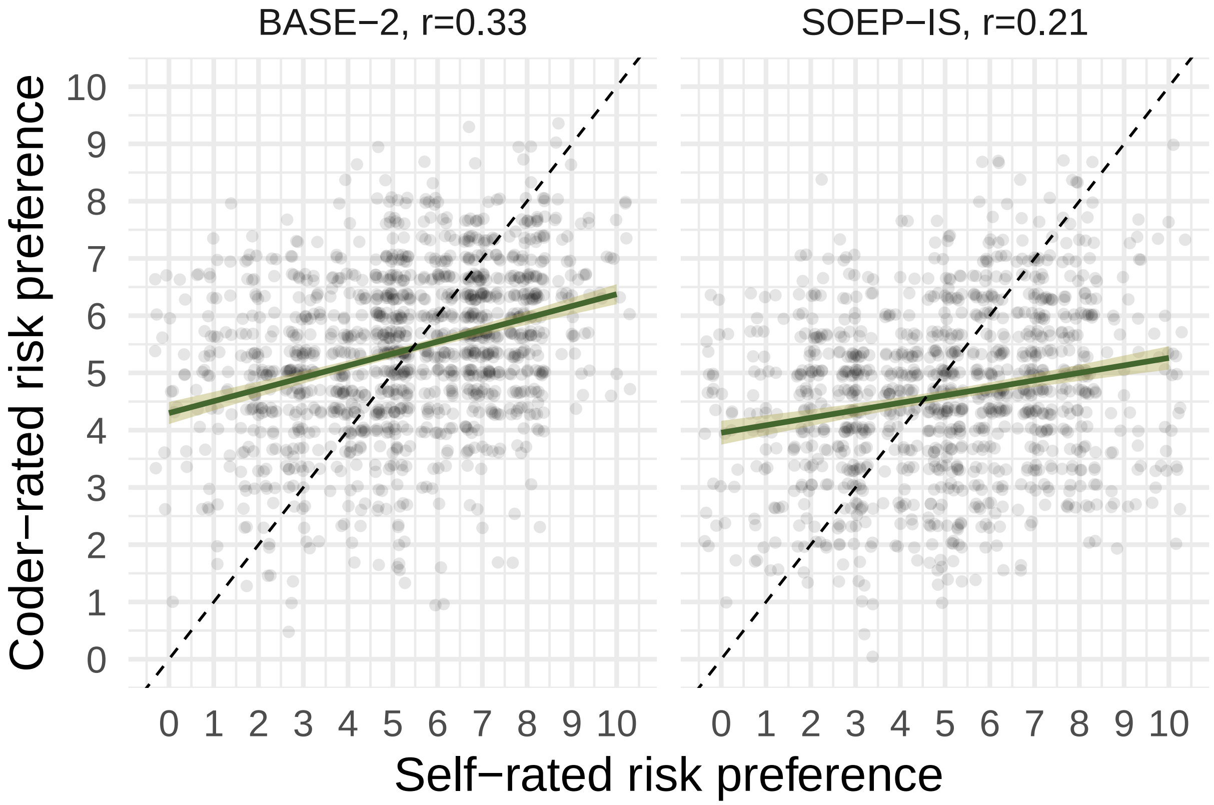

Figure S21: The correlation between coder judgments and stated preferences was higher for BASE-II respondents than for SOEP-IS respondents.

S9.5 Calibration

We wanted to test whether coders were well calibrated. Calibration would be good if coders confidence' was higher when they made more accurate judgments of the respondents' risk preferences. This was the case. The more confident coders were, the larger the correlations between coder ratings and respondent self-reports of risk preferences.

To formally test this, we compared models using LOO-IC. This led to the conclusion that when coders were more confident, the regression slopes of coder ratings on stated preferences were steeper (i.e., coders tended towards the mean less) and the residual standard deviation around the regression line was reduced (i.e., coders were more likely to infer stated preferences accurately).

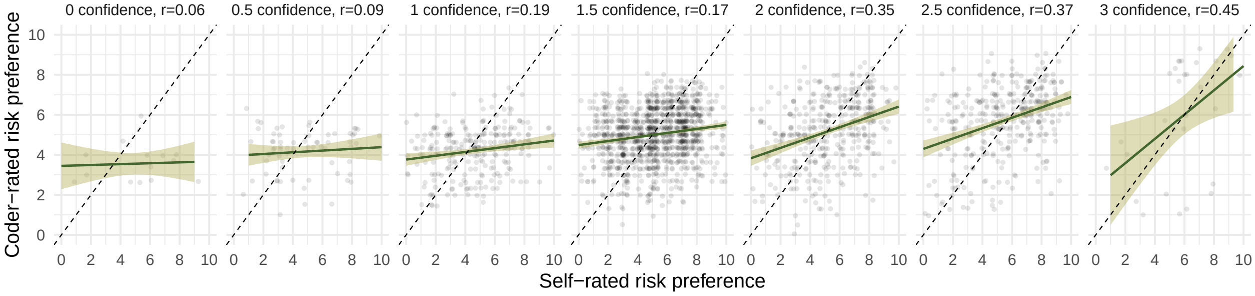

Figure S22: Differences in the correlations according to coder confidence. Correlations between stated preferences and coder judgments increase with confidence.Panels are ordered by rising coder confidence. Scatter plots show tighter fit to the regression line.

Table S20: Calibration model comparison

	LOOIC	SE
m_no_calibration - m_calibration_conf_sigma	17	33
m_no_calibration - m_calibration_conf_interaction	114	38
m_no_calibration - m_calibration_conf_interaction_sigma	164	55
m_calibration_conf_sigma - m_calibration_conf_interaction	97	54
m_calibration_conf_sigma - m_calibration_conf_interaction_sigma	147	44
m_calibration_conf_interaction - m_calibration_conf_interaction_sigma	50	42

Note:
Models were compared using approximative leave-one-out cross-validation (LOO-IC). The first model estimated a simple linear regression. The other three allowed either the slope or the residual to vary by coder confidence, or, as in the case of the best-fitting model (m_conf_interaction_sigma), both.

Table S21: Result from the model preferred by LOO-IC

	Estimated effect [95% CI]
Term	Rated risk preference
**non-varying**	
GRQ	0.06 [0.03;0.09]
GRQ:rating confidence	0.06 [0.04;0.08]
rating confidence	0.11 [0.02;0.20]
sigma: rating confidence	0.20 [0.17;0.23]
**coder (n=9)**	
sd(Intercept)	1.02 [0.60;1.80]
**respondent (n=2293)**	
sd(Intercept)	0.97 [0.92;1.02]

Note:
In this model, we added an interaction between stated risk preference and coder confidence and allowed the residual variation to vary by coder confidence.

S9.6 Only first question

The correlation between coder estimates and stated preferences was smaller when we restricted the data to the responses where only the first question (rs from 0.10 to 0.18 depending on the definition of nonresponse), which focused on explaining the stated preference, had been answered. This correlation should be lower bound, because respondents who only answered the first question also tended to write less for the first question (36 characters) than respondents who answered both (51 characters).

condition	estimate	conf.low	conf.high	n
all	0.27	0.23	0.31	2310
q2_not_codeable	0.18	0.08	0.27	367
q2_no_topic	0.15	0.07	0.23	540
q2_no_text	0.10	-0.05	0.24	178

Note:
Correlations between stated preferences and raters' judgments for four conditions: all data, cases in which the response to the second question was not deemed codeable, cases in which it was deemed to contain no topics, and cases where no text was written in response to the second question at all. 95% confidence intervals are shown.

S9.7 Multiple imputation in case of nonresponse

Table S22: Correlation between stated risk preference and coder ratings after multiple imputation

r	rse	fmi	lower95	upper95
0.3	0.02	0.34	0.26	0.33

Note:
We used the ‘mice‘ package to generate 10 imputations of coder risk preference ratings where missing (usually, because respondents wrote nothing in response to the questions or their text was extremely brief and/or deemed not to include codeable topics. See Figure S10 for details on the multiple imputation. The correlation between stated and rated risk preference was slightly higher than the best estimate before imputation (.27) although the 95% confidence interval included the estimate without imputation. ’rse’ denotes the standard error of the imputed correlation, ’fmi’ denotes the fraction of missing information.

S9.8 Cues

To investigate which cues raters used to inform their judgments of respondents' risk preferences, we employed a lens model analysis and the codings of topics, whether risks were taken or not, and whether risks taken were considered worthwhile.

S9.8.1 Do coders agree on which cues are present?

We evaluated whether coders agreed on the presence of dichotomous cues using Fleiss' kappa, as implemented in the R package `irr`. Coders generally agreed on the common topics and on whether risks were considered worth it. Coders agreed less on the topics `safety` and `crime`, in part because respondents were not always clear about whether they were the victims or perpetrators of crime, and in part because some `safety` topics could also be interpreted as `health` topics. Coders agreed somewhat on whether risks were taken, but did not agree on the alternative answers when they did not think a risk was taken. Coders showed almost no agreement on the specificity of the situation, which is unsurprising given that they were encouraged to use the scale more as a subjective, ordinal response rather than to follow a precise coding scheme.

Table S23: Fleiss' Kappa for major cues

	Fleiss' Kappa			
variable	all	1-3	4-6	7-9
contains_situations_q1	0.13	0.05	0.09	0.16
contains_situations_q2	0.20	-0.02	0.02	0.06
contains_topics_q1	0.89	0.77	0.84	0.86
contains_topics_q2	0.97	0.97	0.98	0.97
risk_worth_it_coded	0.73	0.77	0.70	0.71
risks_taken_or_not	NA	0.12	0.04	0.18
topics_q1_career	0.68	0.85	0.85	0.85
topics_q1_cataclysm	0.00	0.31	0.29	0.61
topics_q1_crime	0.53	0.48	0.48	0.49
topics_q1_gambling	1.00	0.88	0.93	0.89
topics_q1_health	0.73	0.72	0.80	0.75
topics_q1_investments	0.85	0.85	0.90	0.91
topics_q1_relationships	0.87	0.71	0.81	0.77
topics_q1_safety	0.49	0.43	0.62	0.57
topics_q1_sports	0.86	0.91	0.93	0.92
topics_q1_traffic	0.83	0.85	0.94	0.93
topics_q1_travel	0.79	0.88	0.88	0.88
topics_q2_career	0.89	0.84	0.85	0.87
topics_q2_cataclysm	NaN	0.11	0.33	0.60
topics_q2_crime	0.81	0.43	0.63	0.61
topics_q2_gambling	NaN	0.95	0.93	0.93
topics_q2_health	0.71	0.87	0.87	0.84
topics_q2_investments	0.74	0.80	0.87	0.85
topics_q2_relationships	0.80	0.77	0.84	0.78
topics_q2_safety	0.43	0.51	0.72	0.63
topics_q2_sports	0.78	0.88	0.89	0.89
topics_q2_traffic	0.76	0.88	0.92	0.92
topics_q2_travel	0.79	0.84	0.87	0.87

Note:
Table shows Fleiss' Kappa to measure interrater agreement on the presence of certain cues. Cues shown are the major topic categories for Q1 and Q2, specificity of the topic, whether risks were worth it, and whether risks were avoided or taken. Kappas are shown for the set of 50 texts that all coders coded and for the three coder groups 1-3, 4-6, and 7-9. NA/NaN is shown for categories that were never coded for the first 50 texts.

S9.8.2 Which cues vary enough?

To exclude cues that were too rare to explain judgments substantially, we excluded coded dichotomous cues with frequencies lower than 1% or higher than 99%. Specifically, we applied a threshold of a standard deviation of at least .10 (equivalent to a mean frequency of .01 or .99) to a priori exclude cues that are too rare to matter.

Table S24: Included cues

var	freq	sd
contains__situations__q1__multiple__concrete__situations	0.05	0.22
contains__situations__q1__specific__topic	0.21	0.41
contains__situations__q1__unknown__time__and__place__but__concrete__behaviour	0.26	0.44
contains__situations__q1__vague__topic	0.08	0.27
contains__situations__q2__specific__topic	0.11	0.31
contains__situations__q2__unknown__time__and__place__but__concrete__behaviour	0.28	0.45
contains__situations__q2__vague__topic	0.03	0.16
contains__topics__q1	0.90	0.30
contains__topics__q2	0.71	0.45
health__q1__operation	0.01	0.11
health__q1__other	0.04	0.20
health__q2__operation	0.05	0.21
health__q2__other	0.04	0.20
investments__q1__bought__home	0.03	0.17
investments__q1__investment	0.08	0.27
investments__q1__other	0.15	0.35
investments__q2__bought__home	0.01	0.12
investments__q2__investment	0.06	0.24
investments__q2__other	0.08	0.27
meaningful__entry__q1__nothing	0.04	0.19
meaningful__entry__q1__nothing__concrete	0.02	0.13
meaningful__entry__q2__nothing	0.19	0.39
number__topics__q1	0.00	1.00
number__topics__q2	0.00	1.00
relationships__q1__children__general	0.02	0.13
relationships__q1__conflicts	0.02	0.15
relationships__q1__moving	0.04	0.20
relationships__q1__other	0.09	0.28
relationships__q1__speaking__out	0.01	0.11
relationships__q2__children__general	0.01	0.11
relationships__q2__conflicts	0.02	0.14
relationships__q2__moving	0.03	0.16
relationships__q2__other	0.07	0.25
risk__worth__it__coded__cant__tell__yet	0.02	0.14
risk__worth__it__coded__mixed	0.05	0.21
risk__worth__it__coded__no__real__answer	0.03	0.16
risk__worth__it__coded__not__worth__it	0.04	0.21
risk__worth__it__coded__several	0.01	0.12
risk__worth__it__coded__worth__it	0.30	0.46
risks__taken__or__not__no__avoided	0.01	0.12
risks__taken__or__not__no__others	0.01	0.11

risks_taken_or_not_unclear	0.29	0.45
risks_taken_or_not_yes	0.47	0.50
safety_q1_construction_gardening	0.01	0.11
safety_q1_expose_to_criminals	0.02	0.12
safety_q1_frailty	0.02	0.15
safety_q1_other	0.06	0.23
safety_q2_construction_gardening	0.02	0.15
safety_q2_frailty	0.02	0.15
safety_q2_other	0.02	0.15
sports_q1_mountaineering	0.03	0.16
sports_q1_other	0.07	0.25
sports_q2_mountaineering	0.02	0.14
sports_q2_other	0.04	0.20
topics_q1_career	0.18	0.39
topics_q1_gambling	0.04	0.20
topics_q1_health	0.08	0.27
topics_q1_investments	0.27	0.44
topics_q1_other	0.08	0.28
topics_q1_relationships	0.22	0.42
topics_q1_safety	0.14	0.34
topics_q1_sports	0.12	0.33
topics_q1_traffic	0.18	0.39
topics_q1_travel	0.10	0.30
topics_q2_career	0.15	0.35
topics_q2_crime	0.01	0.10
topics_q2_gambling	0.03	0.18
topics_q2_health	0.11	0.32
topics_q2_investments	0.17	0.38
topics_q2_other	0.04	0.19
topics_q2_relationships	0.17	0.37
topics_q2_safety	0.09	0.29
topics_q2_sports	0.08	0.28
topics_q2_traffic	0.15	0.35
topics_q2_travel	0.10	0.30
traffic_q1_bicycling	0.04	0.19
traffic_q1_car	0.07	0.26
traffic_q1_motorcycle	0.01	0.12
traffic_q1_other	0.06	0.24
traffic_q2_bicycling	0.04	0.20
traffic_q2_car	0.07	0.26
traffic_q2_other	0.02	0.14

Note:
All nondichotomous cues and dichotomous cues with frequencies between 1% and 99% were included.

S9.8.3 Lens model

We then performed two parallel multiple regression analyses to predict judgments and stated preferences from all cues simultaneously. We used `brms` and specified a lasso prior with one degree of freedom to regularise coefficients (Bürkner, 2017). One regression predicted the coder rating, the judgment; one predicted the stated preference by the respondent, the criterion.

Based on the regression models, we correlated actual judgments, actual stated preferences, the judgments predicted by the regression, and the stated preferences predicted by the regression to derive the coefficients explained below. Additionally, we estimated a leave-one-out-adjusted R2 to further reduce overfitting to the data.

- r_a *Achievement*: Correlation between actual judgment and actual criterion
- R_S *Consistency*: Correlation between predicted judgment and actual judgment (i.e., do coders use the cues consistently?)
- R_E *Predictability*: Correlation between predicted criterion and actual criterion (i.e., how well can the criterion be predicted from the available cues?)
- G *Knowledge* (Matching index): Correlation between predicted judgment and predicted criterion (i.e., does the judge use cues according to their validity?)
- C *Configurality*: Correlation between the residuals of predicted judgment and predicted criterion (i.e., greater if there is evidence for interactions between cues)

Table S25: Lens model estimates

index	r
achievement	0.27
consistency	0.67
predictability	0.36
knowledge	0.75
configurality	0.15
predictability_loo	0.31
consistency_loo	0.64

We found that coder ratings correlated .61 with the prediction by the judgment regression, which means that coders used the available cues fairly consistently. The available cues could predict the stated preference with a correlation of .37. These results could have been slightly inflated by overfitting in spite of the lasso prior meant to guard against it. Leave-one-out-adjusted multiple correlations were only slightly lower (.60 and .31). Coder accuracy (.27) was very close to the leave-one-out-adjusted predictability, showing that coders made generally good use of the cues that they coded.

The correlation between coder judgments and respondents' stated preferences (achievement) is reproducible from the coefficients explained above:

$$r_a = G * R_E * R_S + C * \sqrt{1 - R_E^2} * \sqrt{1 - R_S^2}$$

Result: 0.28

S9.8.3.1 Regression coefficients

Table S26: Predicting rater judgments and respondents' stated preferences from the same cues

	Judgment (cue utilization)			Stated (cue validity)		
term	estimate	lower	upper	estimate	lower	upper
risks_taken_or_not_no_avoided	-0.59	-0.81	-0.35	-0.22	-0.49	0.00
contains_situations_q1_vague_topic	-0.55	-0.66	-0.44	0.03	-0.06	0.12
topics_q2_crime	0.44	0.18	0.70	0.03	-0.10	0.19
traffic_q1_motorcycle	0.42	0.17	0.68	0.11	-0.04	0.34
contains_situations_q2_vague_topic	-0.41	-0.58	-0.24	0.01	-0.10	0.13
risks_taken_or_not_yes	0.36	0.28	0.45	0.16	0.07	0.25
topics_q2_sports	0.36	0.20	0.52	0.17	0.03	0.32
topics_q2_gambling	0.32	0.15	0.50	0.08	-0.04	0.24
risks_taken_or_not_no_others	-0.31	-0.57	-0.06	0.00	-0.14	0.13
meaningful_entry_q2_nothing	-0.31	-0.41	-0.21	-0.11	-0.23	0.00
health_q2_other	-0.26	-0.44	-0.07	-0.07	-0.22	0.05
risk_worth_it_coded_worth_it	0.24	0.17	0.31	0.09	0.01	0.17
topics_q1_investments	0.23	0.09	0.37	-0.06	-0.18	0.04
relationships_q1_conflicts	-0.22	-0.42	-0.03	-0.05	-0.20	0.07
relationships_q1_other	-0.21	-0.35	-0.09	0.00	-0.09	0.09
sports_q1_mountaineering	0.21	0.01	0.42	0.05	-0.07	0.20
investments_q1_other	-0.20	-0.35	-0.05	-0.18	-0.32	-0.03
safety_q1_expose_to_criminals	-0.20	-0.43	0.00	-0.10	-0.30	0.05
topics_q2_other	-0.20	-0.34	-0.06	0.01	-0.10	0.12
safety_q2_construction_gardening	0.19	0.01	0.40	0.01	-0.11	0.15
safety_q2_other	-0.19	-0.40	0.00	-0.02	-0.15	0.09
contains_situations_q2_specific_topic	-0.19	-0.28	-0.09	0.04	-0.04	0.13
topics_q2_health	0.19	0.04	0.34	-0.03	-0.14	0.06
sports_q2_other	-0.18	-0.38	0.00	0.00	-0.12	0.11
contains_situations_q2 unknown_time_and_place concrete_behaviour	0.18	0.11	0.25	0.02	-0.04	0.10
traffic_q2_bicycling	-0.17	-0.34	-0.01	-0.01	-0.12	0.09
contains_situations_q1 unknown_time_and_place concrete_behaviour	0.17	0.09	0.24	0.04	-0.03	0.12
traffic_q1_other	-0.16	-0.34	0.00	-0.18	-0.34	-0.03
topics_q2_travel	0.16	0.06	0.27	-0.07	-0.17	0.02
number_topics_q2	0.16	0.10	0.22	0.12	0.06	0.18
topics_q1_sports	0.16	0.02	0.29	0.09	-0.02	0.22
risk_worth_it_coded_several	0.14	-0.03	0.36	-0.04	-0.20	0.08
risk_worth_it_coded_cant_tell_yet	0.14	-0.02	0.33	0.03	-0.09	0.17
contains_situations_q1 multiple_concrete_situations	0.14	0.01	0.26	-0.02	-0.13	0.08
topics_q1_traffic	-0.13	-0.27	0.01	-0.05	-0.17	0.03
health_q1_other	-0.11	-0.28	0.03	-0.10	-0.27	0.03
topics_q1_safety	-0.11	-0.23	0.00	-0.01	-0.10	0.08
investments_q2_bought_home	0.11	-0.07	0.31	-0.04	-0.20	0.09
traffic_q2_other	-0.10	-0.30	0.06	0.00	-0.12	0.13

investments_q1_investment	0.10	-0.05	0.26	-0.14	-0.30	0.00
traffic_q1_car	-0.10	-0.26	0.04	-0.01	-0.11	0.10
safety_q1_construction_gardening	0.10	-0.09	0.31	0.09	-0.05	0.29
relationships_q2_other	-0.09	-0.23	0.02	-0.02	-0.12	0.07
relationships_q1_moving	0.09	-0.04	0.24	0.05	-0.05	0.19
topics_q1_career	0.09	0.01	0.18	0.09	0.00	0.18
topics_q2_safety	0.08	-0.04	0.23	0.00	-0.10	0.09
relationships_q2_moving	-0.08	-0.24	0.06	0.00	-0.11	0.11
contains_situations_q1_specific_topic	-0.08	-0.15	0.00	-0.05	-0.14	0.02
risk_worth_it_coded_no_real_answer	-0.08	-0.23	0.05	-0.21	-0.42	-0.03
meaningful_entry_q1_nothing_concrete	0.08	-0.08	0.26	0.01	-0.11	0.15
topics_q1_relationships	0.07	-0.02	0.18	0.02	-0.05	0.11
health_q1_operation	-0.07	-0.29	0.11	0.06	-0.06	0.26
topics_q1_other	-0.07	-0.18	0.02	0.06	-0.03	0.18
topics_q1_gambling	0.07	-0.05	0.21	0.02	-0.09	0.13
topics_q2_career	0.06	-0.03	0.15	0.01	-0.07	0.10
topics_q2_investments	0.06	-0.05	0.18	0.05	-0.04	0.15
relationships_q2_conflicts	0.05	-0.11	0.23	-0.03	-0.17	0.10
relationships_q1_children_general	-0.05	-0.23	0.10	-0.05	-0.22	0.08
relationships_q2_children_general	0.05	-0.12	0.25	0.01	-0.12	0.16
topics_q1_travel	0.05	-0.04	0.15	0.05	-0.03	0.15
topics_q2_traffic	0.05	-0.06	0.18	0.01	-0.08	0.10
number_topics_q1	0.05	0.00	0.10	-0.01	-0.06	0.03
investments_q2_investment	0.05	-0.08	0.18	0.07	-0.04	0.19
investments_q1_bought_home	-0.05	-0.21	0.10	0.02	-0.09	0.15
risk_worth_it_coded_mixed	0.04	-0.07	0.16	0.00	-0.11	0.10
safety_q1_frailty	-0.04	-0.21	0.11	-0.03	-0.16	0.09
traffic_q2_car	-0.04	-0.17	0.08	-0.03	-0.14	0.07
safety_q2_frailty	-0.04	-0.22	0.12	-0.11	-0.32	0.03
topics_q2_relationships	0.03	-0.07	0.14	-0.02	-0.11	0.06
risks_taken_or_not_unclear	0.03	-0.05	0.12	0.05	-0.02	0.15
investments_q2_other	0.03	-0.09	0.16	0.03	-0.06	0.14
relationships_q1_speaking_out	0.03	-0.14	0.20	0.03	-0.10	0.18
safety_q1_other	0.03	-0.10	0.17	0.03	-0.07	0.14
sports_q1_other	-0.02	-0.17	0.11	0.08	-0.03	0.23
risk_worth_it_coded_not_worth_it	0.02	-0.08	0.14	-0.04	-0.17	0.06
topics_q1_health	-0.02	-0.14	0.10	-0.03	-0.15	0.07
health_q2_operation	0.02	-0.13	0.17	-0.01	-0.12	0.11
contains_topics_q2	-0.02	-0.13	0.09	0.07	-0.03	0.20
sports_q2_mountaineering	0.01	-0.17	0.20	0.00	-0.13	0.13
traffic_q1_bicycling	-0.01	-0.16	0.13	-0.04	-0.17	0.08
meaningful_entry_q1_nothing	0.01	-0.12	0.15	0.00	-0.11	0.12
contains_topics_q1	0.01	-0.11	0.13	0.00	-0.11	0.10

Note:
Regression estimates and 95% credible intervals as estimated in a Lasso regression.

S10 Supplementary References

1. Tynan, M. The Domain-Specific Risk-Taking Scale lacks convergence with alternative risk-taking propensity measures. (Iowa State University, 2018). doi:%5B10.31274/etd-180810-6107](https://doi.org/10.31274/etd-180810-6107).
2. Charness, G., Garcia, T., Offerman, T. & Villeval, M. C. Do measures of risk attitude in the laboratory predict behaviour under risk in and outside of the laboratory? Journal of Risk and Uncertainty (2020) doi:%5B10.1007/s11166-020-09325-6](https://doi.org/10.1007/s11166-020-09325-6).
3. Pedroni, A. et al. The risk elicitation puzzle. Nature Human Behaviour (2017) doi:%5B10.1038/s41562-017-0219-x](https://doi.org/10.1038/s41562-017-0219-x).
4. Frey, R., Pedroni, A., Mata, R., Rieskamp, J. & Hertwig, R. Risk preference shares the psychometric structure of major psychological traits. Science advances 3, e1701381 (2017).
5. Lönnqvist, J.-E., Verkasalo, M., Walkowitz, G. & Wichardt, P. C. Measuring individual risk attitudes in the lab: Task or ask? An empirical comparison. J. Econ. Behav. Organ. 119, 254–266 (2015).
6. Coppola, M. Eliciting risk-preferences in socio-economic surveys: How do different measures perform? J. Socio Econ. 48, 1–10 (2014).
7. Chuang, Y. & Schechter, L. Stability of experimental and survey measures of risk, time, and social preferences: A review and some new results. J. Dev. Econ. 117, 151–170 (2015).
8. Galizzi, M. M., Machado, S. R. & Miniaci, R. Temporal Stability, Cross-Validity, and External Validity of Risk Preferences Measures: Experimental Evidence from a UK Representative Sample. Social Science Research Network (2016) doi:%5B10.2139/ssrn.2822613](https://doi.org/10.2139/ssrn.2822613).
9. Mata, R., Frey, R., Richter, D., Schupp, J. & Hertwig, R. Risk Preference: A View from Psychology. J. Econ. Perspect. 32, 155–172 (2018).
10. Hertwig, R., Wulff, D. U. & Mata, R. Three gaps and what they may mean for risk preference. Philos. Trans. R. Soc. Lond. B Biol. Sci. (2019) doi:%5B10.1098/rstb.2018.0140](https://doi.org/10.1098/rstb.2018.0140).
11. Charness, G., Gneezy, U. & Imas, A. Experimental methods: Eliciting risk preferences. J. Econ. Behav. Organ. 87, 43–51 (2013).
12. Harrison, J. D., Young, J. M., Butow, P., Salkeld, G. & Solomon, M. J. Is it worth the risk? A systematic review of instruments that measure risk propensity for use in the health setting. Soc. Sci. Med. 60, 1385–1396 (2005).
13. Bran, A. & Vaidis, D. C. Assessing risk-taking: what to measure and how to measure it. J. Risk Res. 1–14 (2019) doi:%5B10.1080/13669877.2019.1591489](https://doi.org/10.1080/13669877.2019.1591489).
14. Szrek, H., Chao, L.-W., Ramlagan, S. & Peltzer, K. Predicting (un)healthy behaviour: A comparison of risk-taking propensity measures. Judgm. Decis. Mak. 7, 716–727 (2012).
15. Falk, A. et al. Global Evidence on Economic Preferences. Q. J. Econ. 133, 1645–1692 (2018).
16. Beauchamp, J. P., Cesarini, D. & Johannesson, M. The psychometric and empirical properties of measures of risk preferences. J. Risk Uncertain. 54, 203–237 (2017).
17. Dohmen, T. et al. Individual Risk Attitudes: Measurement, Determinants, and Behavioral Consequences. J. Eur. Econ. Assoc. 9, 522–550 (2011).
18. Highhouse, S., Nye, C. D., Zhang, D. C. & Rada, T. B. Structure of the DOSPERT: is there evidence for a general risk factor? Journal of Behavioral Decision Making 30, 400–406 (2017).
19. Mõttus, R., Bates, T., Condon, D. M., Mroczek, D. & Revelle, W. Your personality data can do more: Items provide leverage for explaining the variance and co-variance of life outcomes. PsyArXiv (2017).
20. Mõttus, R., Kandler, C., Bleidorn, W., Riemann, R. & McCrae, R. R. Personality traits below facets: The consensual validity, longitudinal stability, heritability, and utility of personality nuances. J. Pers. Soc. Psychol. 112, 474–490 (2017).
21. Revelle, W. et al. Web and phone based data collection using planned missing designs. in The SAGE Handbook of Online Research Methods (eds. Fielding, N. G., Lee, R. M. & Blank, G.) (SAGE, 2016).
22. Conway, J. R., Lex, A. & Gehlenborg, N. UpSetR: an R package for the visualization of intersecting sets and their properties. Bioinformatics 33, 2938–2940 (2017).
23. Bürkner, P.-C. brms: An R package for Bayesian multilevel models using Stan. J. Stat. Softw. 80, (2017).
24. Rosseel, Y. lavaan: An R Package for Structural Equation Modeling. J. Stat. Softw. 48, 1–36 (2012).
